# Seeding and Harvest: A Framework for Unsupervised Feature Selection Problems

**DOI:** 10.3390/s130100292

**Published:** 2012-12-27

**Authors:** Gang Chen, Yuanli Cai, Juan Shi

**Affiliations:** School of Electronic and Information Engineering, Xi'an Jiaotong University, No.28, Xianning West Road, Xi'an 710049, China; E-Mails: ylicai@mail.xjtu.edu.cn (Y.C.); shijuan4258@gmail.com (J.S.)

**Keywords:** feature selection, seeding and harvest, noise injection

## Abstract

Feature selection, also known as attribute selection, is the technique of selecting a subset of relevant features for building robust object models. It is becoming more and more important for large-scale sensors applications with AI capabilities. The core idea of this paper is derived from a straightforward and intuitive principle saying that, if a feature subset (pattern) has more representativeness, it should be more self-organized, and as a result it should be more insensitive to artificially seeded noise points. In the light of this heuristic finding, we established the whole set of theoretical principles, based on which we proposed a two-stage framework to evaluate the relative importance of feature subsets, called seeding and harvest (S&H for short). At the first stage, we inject a number of artificial noise points into the original dataset; then at the second stage, we resort to an outlier detector to identify them under various feature patterns. The more precisely the seeded points can be extracted under a particular feature pattern, the more valuable and important the corresponding feature pattern should be. Besides, we compared our method with several state-of-the-art feature selection methods on a number of real-life datasets. The experiment results significantly confirm that our method can accomplish feature reduction tasks with high accuracy as well as low computing complexity.

## 1. Introduction

There are more and more sensor applications requiring artificial intelligence (AI), machine learning and data mining technologies to identify new, potential and useful knowledge from datasets [1], which are becoming larger and larger in real life along with the emergence of internet [2] and bio-informatics [3]. Thus, data preprocessing is becoming increasingly crucial, especially the data reduction process, by which the AI modules of sensors could produce their results within acceptable computing time.

**Figure 1.**
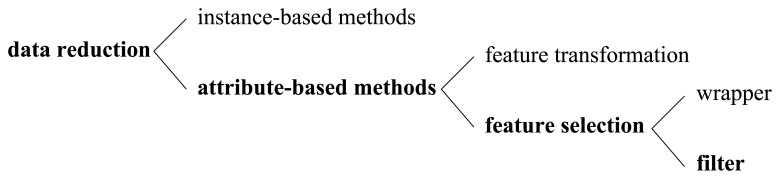
Categories of data reduction methods. The categories that our method belongs to are in boldface.

As illustrated in Figure 1, there are mainly two categories of data reduction methodologies, which are instance-based ones and attribute-based (feature-based) ones.

Instance-based data reduction methods like various sampling techniques have been studied thoroughly [4,5], whose main purpose is to reduce total entities in a dataset. However, in many applications such as decision support, pattern recognition and financial forecasts [6], we cannot solve the whole problem only relying on instance reduction, because there are often hundreds, thousands, even millions of attributes in real-life datasets, and most of them may be irrelevant or redundant. That is to say, the bottleneck here lies in the number of features, instead of the number of instances. Meanwhile, as we know, high dimensionality of data may cause the “curse of dimensionality” problem [7]. Therefore, attribute-based technologies deserve to be studied deeply to find more effective and more efficient methods, with which the total features of a dataset can be dramatically reduced, thereby more sophisticated AI algorithms could become feasible on high-dimensional datasets.

Refer to the third column of Figure 1, attribute-based data reduction methods [8] fall into two general categories. One is feature transformation, and the other is feature selection. They are distinct from each other in whether new features are produced or not. Feature transformation methods like principal component analysis (PCA) [9] and factor analysis (FA) transform original features into some new features and factors respectively, which are probably difficult to interpret for human beings [10]. In contrast, the methodology adopted by feature selection methods is trying to search for the most valuable feature subset heuristically (searcher) under certain predefined feature subset evaluation criterion (evaluator). Why is the searcher required? As we have pointed out, the number of features is often huge, not to mention the number of possible feature subsets, so it is impractical to impose the evaluator on each possible feature subset to get the best one [5]. For instance, if we have a dataset of *d* features, the number of possible feature subsets will reach 2*^d^*, which will become prohibitively large even with a moderately increasing *d*. So, cooperating with the evaluator, a heuristic searcher is often required and employed in feature selection tasks. Greedy hill climbing and best first search are two classical search methods adopted widely [11]. Meanwhile, some sophisticated methods such as genetic search [12] and fuzzy reasoning search [13] can also be employed.

According to what kind of evaluator has been adopted, a feature selection methodology can be further categorized into a wrapper or a filter, which are distinct from each other in whether a specific AI algorithm is required as the measure of relative importance of different feature subsets (the last column of Figure 1). Specifically speaking, in a wrapper method, an AI algorithm must be predefined, and the performance of this AI algorithm under a particular feature subset is seen as the measurement of the relative importance of this feature subset. For example, if the dataset is going to be mined by C4.5 classification algorithm [14], then the relative importance of a feature subset could be evaluated according to the accuracy of C4.5 algorithm performed under that feature subset. Every coin has two sides: on one hand, wrappers can achieve good results if the feature-reduced dataset is going to feed the same AI algorithm that has already been employed in the evaluator. But on the other hand, because of losing generality, wrappers are prone to bad performance when the feature-reduced dataset is going to feed any other AI algorithm that is different from the one employed in the evaluator. Moreover, wrapper-based methods are often too slow to employ in large scale applications, especially in circumstances where sophisticated AI algorithms are involved. In contrast to wrappers, filters are independent of any specific AI algorithm by taking advantage of some general criteria to evaluate the feature subsets. Since filters are more adaptive and efficient, they are becoming more and more popular in high-dimensional AI and data mining problems. In this paper, to tackle the feature reduction problems, we proposed a filter-based feature selection method, which belongs to the boldface categories in Figure 1.

From another aspect of whether the label (class) information is considered, feature reduction methodologies can also be classified into supervised and unsupervised ones. As we see, the label information may be difficult to access in many applications, and there are more and more datasets given without label information. Hence in this paper, we will concentrate on the unsupervised methods. As we can infer, because supervised methods take the auxiliary label information into consideration, they are probably more suitable for classification tasks, while unsupervised methods are prone to be more suitable for clustering tasks [15]. Thus, most of the theoretical analysis, practical examples, and performance evaluations in this paper are clustering-oriented.

Generally speaking, in this paper, we proposed a flexible framework called S&H, which is capable of ordering feature subsets according to their relative importance (sorter). To cooperate with the sorter, we improved the traditional heuristic searching methodologies into order-based ones, which can be called ordinal searchers. The above two components—sorter and ordinal searcher—compose our main structure to handle the feature selection problem, which is distinct from the traditional “evaluator and searcher” structure, as we concentrate on “orders” but not “values”. That property makes our structure more sensible and straightforward, because the underlying purpose of feature selection is just to find out the best feature pattern, but not to answer how superior that feature pattern is quantitatively.

As stated above, our S&H sorter framework was initially inspired by a simple intuitive principle, namely, if a feature subset has more representativeness, it should be more self-organized, and as a result it should be more insensitive to artificially injected noise points. That is to say, our S&H sorter can be divided into two main stages. The first stage is called “seeding”, and the second one is “harvest”. At the seeding stage, we inject some artificial noise points into the dataset, and in the harvest stage, we resort to a uniformly partitioning-based outlier detector [16] to identify them from the original dataset. From this novel point of view, the S&H framework virtually turns the feature subset ordering problem into outlier detection problem—the relative importance of feature subsets can be assessed and ordered according to how precisely the artificial noises (outliers) can be detected under these feature subsets. One may wonder, why we call S&H a framework? As one can infer, S&H is not confined to specific kinds of seeder and harvester. That is, other kinds of noise generating (seeder) and outlier detection (harvester) algorithms can also be adopted to construct a new S&H implementation. For instance, instead of the random injection methodology we adopted, people can also employ some kind of deterministic grid point injection methodology in the seeding stage. Analogously, in the harvest stage, a lot of other off-the-shelf outlier detection methods can also be employed, such as LOF [17] and iForest [18]. Although our S&H framework is flexible to have plenty of variants, to be concrete, only one S&H implementation will be studied thoroughly in this paper, where the uniformly distributing-based seeder and uniformly partitioning-based harvester will be adopted.

Although derived from an intuitive principle, our methodology is based on solid theoretical foundations. The key points are listed as follows:

1. We modeled the feature-selected clustering problem into a rigorous optimization form in mathematics.
2. We proposed the concept of coverability, which was proved to be an intrinsic property of a certain dataset.
3. We showed that solving the feature selection problem is equal to finding the specific feature pattern, under which the dataset exhibits the smallest coverability.
4. We found the correlation between coverability and the probability with which the seeded points can be detected correctly.
5. We eventually concluded that solving the feature selection problem is equal to finding the specific feature subset in which the seeded points can be extracted most exactly.

This paper is organized as follows: In Section 2, we review some related work. In Section 3, we present our main principles involved. The practical interpretation of the theories is given in Section 4, with some important considerations in practice. In Section 5, we describe the implementation of our methodology in detail, and provide the main algorithms in pseudo-code. The comparison experiments on extensive datasets are analyzed in Section 6; and finally, our conclusions are presented in Section 7.

## 2. Related Work

This section briefly reviews the state-of-the-art feature selection algorithms, which can be categorized according to a number of criteria as we have illustrated in Figure 1. Unless stated otherwise, we only focus our attention on filter-based feature selection methods.

A rather simple attribute ranking method is the information gain [19] (IG) method. It is based on the concept of entropy. Equation (1) and Equation (2) give the entropy [20] of the class before and after observing the attribute, where *a* stands for an attribute and *c* stands for a class.

(1) $$H(C)=-\sum_{c\in C}p(c)\log_{2}p(c),$$

(2) $$H(C|A)=-\sum_{a\in A}p(a)-\sum_{c\in C}p(c|a)\log_{2}p(c|a).$$

Thus, we get the information gain (IG) for attribute *A_i_* from Equation (3)

(3) $$IG_{i}=H(C)-H(C|A_{i}).$$

Inspired by IG, people developed a lot of more sophisticated information-based methods. Liu et al. introduced the dynamic mutual information method [21], and Yan *et al.* introduced a correntropy-based method [22] recently.

Relief [23,24] is a typical instance-based attribute ranking method. It works by randomly sampling an instance and characterize its nearest neighbours. Recently, Janez has extended it for attribute subset evaluation [25].

CFS [5,26] was the first of the methods that evaluate subsets of attributes rather than individual attributes [19]. Its main hypothesis is that a good feature subset is the one that contains features highly correlated with the class, yet uncorrelated with each other. This heuristic assigns high scores to subsets containing attributes that are highly correlated with the class and have low inter-correlation with each other. The following equation:

(4) $${\text{Merit}}_{S}=\frac{k\bar{r_{cf}}}{\sqrt{k+k(k-1)\bar{r_{\text{cff}}}}},$$

gives the merit of an attribute subset, where $\bar{r_{cf}}$ is the average feature-class correlation, and $\bar{r_{ff}}$ is the average feature-feature inter-correlation. *Merit_S_* denotes the heuristic “merit” of a feature subset *S* containing *k* features. Compared with other methods we have mentioned, CFS chooses fewer features, is faster and produces smaller trees [19].

Consistency-based methods [27,28] look for combinations of attributes whose values divide the data into subsets containing a strong single class majority. Usually the search is biased in favor of small feature subsets with high class consistency [19].

All the above are supervised feature selection methods. Compared with them, the unsupervised methods do not need class labels. Next, we will review some unsupervised methods.

A common category of unsupervised feature selection methodology is the one based on various clustering technologies. For example, Dy and Brodley proposed a cluster-based method [29], which explores the feature selection problem through FSSEM (Feature Subset Selection using Expectation-Maximization (EM) clustering) and two different performance criteria for evaluating candidate feature subsets: scatter separability and maximum likelihood. Hong et al. proposed a feature selection algorithm for unsupervised clustering [30], which combines the clustering ensembles method and the population-based incremental learning algorithm. The main idea of this algorithm is to search for a subset of all features such that the clustering algorithm trained on this feature subset can achieve the most similar clustering solution to the one obtained by an ensemble learning algorithm. With the idea of selecting those features such that the multi-cluster structure of the data can be best preserved, Cai et al. proposed their method recently [31].

There also exist other kinds of unsupervised methods. As we know, some transformation-based methods like PCA and FA are statistical unsupervised methods, which have been discussed in Section 1. Besides them, a spectrum-based method [32] is proposed by Zhao and Liu. Moreover, Mitra *et al.* proposed an unsupervised feature selection method using feature similarity [33]. In summary, the unsupervised methods evaluate feature relevance by the capability of keeping certain properties of original data [21].

Generally speaking, the most significant difference between this work and other unsupervised methods resides in that, we are the first to resort to outlier detection technologies to study feature selection problems. This purpose is achieved by means of our fundamental theories, which will be covered in the next section.

## 3. Main Principle

Before introducing our theories, we believe that we should demonstrate the importance of feature selection through a simple but concrete example.

**Figure 2.**
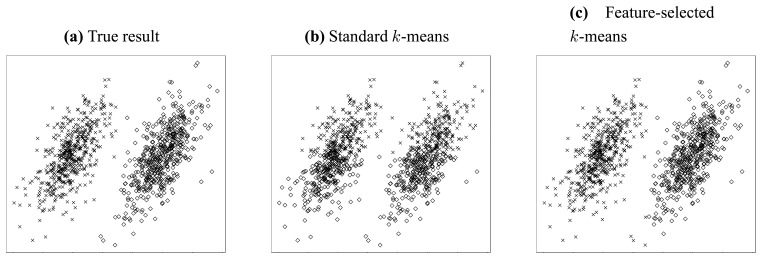
The effect of feature selection, where the only difference between the two clusters lies in the fluctuation of their horizontal means.

Let us consider the simple clustering problem illustrated in Figure 2. In this problem, two independent jointly Gaussian clusters are generated, and they are distinct from each other only in their horizontal means (Figure 2(a)).

Thus, we can conjecture that the most valuable information resides in the horizontal dimension. To clarify this point, we try to cluster this dataset using standard 2-means method [34]. Figure 2(b) gives the result when both features (dimensions) are considered, while Figure 2(c) shows the result when only the horizontal feature is employed. It is obvious from above two figures that the accuracy can be improved dramatically if somehow we can know that the horizontal feature is more valuable and thereby apply clustering using that feature only. Through this simple but explicit example, we see that feature selection is so important that it is indispensable for a lot of clustering applications, especially in high-dimensional circumstances.

Because of the intuitive and heuristic natures of our methodology, it would be much more straightforward to explain through visible examples other than pure theories. Thus, in the following, as a beginning, we will represent the core ideas of our methodology through the analysis on a simple synthetic multidimensional dataset.

### 3.1. The Intuitions Derived from A Simple Example

**Figure 3.**
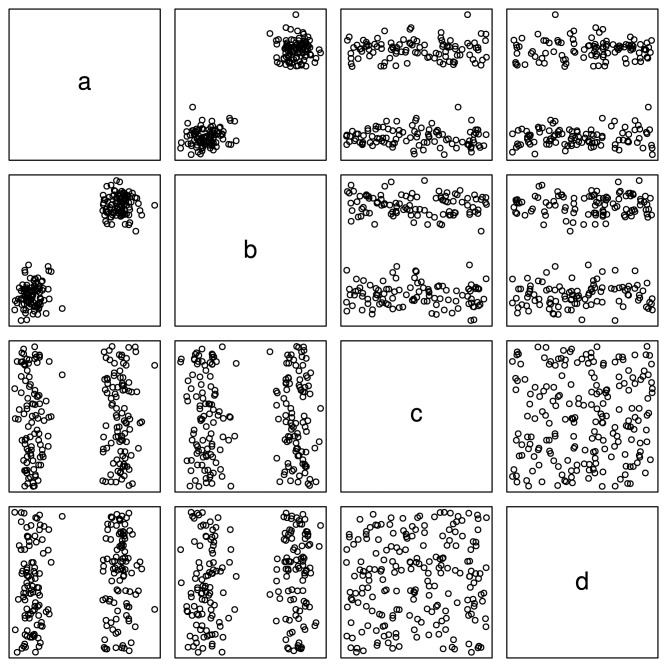
Scatter plots for the synthetic dataset consisting of 4 attributes: *a*, *b*, *c*, and *d*.

Let us inspect the synthetic dataset shown in Figure 3.

**Figure 4.**
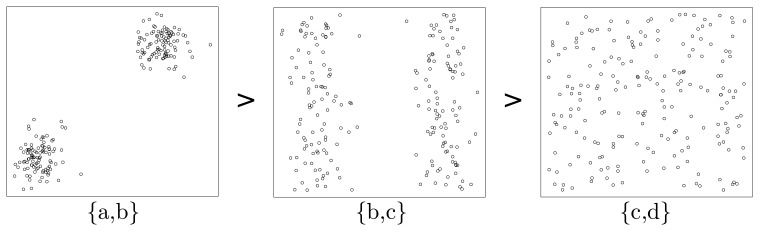
The significance (relative importance) order of attribute subsets—{*a*, *b*}, {*b*, *c*} and {*c*, *d*}.

This figure gives the linked two-dimensional scatter plots of our synthetic multidimensional dataset consisting of 4 independent attributes labeled *a*, *b*, *c*, and *d*, where *a* and *b* are normally distributed while *c* and *d* are uniformly distributed. Two more things should be pointed out here. First, the linked two-dimensional scatter plots are a display technique, by which multidimensional observations can be represented in two dimensions [35]. For example, Figure 3 shows two-dimensional scatter plots for pairs of these attributes organized as a 4 × 4 array. Second, our method does not rely on any prior assumption of underlying distributions of attributes. We adopt the normal and uniform distributions here to make this example as evident as possible. Therefore, let us inspect three typical attribute subsets—{*a*, *b*}, {*b*, *c*} and {*c*, *d*} of this dataset, and we can easily find out that, in the subplot of attribute *a* and *b* (the cell in the cross of the second row and the first column of Figure 3), there are two normally distributed clusters in the top right corner and lower left corner, while in the subplots of attribute subset {*b*, *c*} (the cell in the cross of the third row and the second column of Figure 3) and {*c*, *d*} (the cell in the cross of the fourth row and the third column of Figure 3), there are two belt-shaped clusters and no significant cluster respectively. To make it clearer, we extract the subplots of the above three attribute subsets and list them in Figure 4.

Now, let us inspect the fundamental problem of ordering these three attribute subsets ({*a*, *b*}, {*b*, *c*} and {*c*, *d*}) according to their merits (relative importance). As one may conjecture that, the relative importance of attribute subsets can be qualitatively assessed by means of the entropy criterion. The concept of entropy is involved in the information theory. Roughly speaking, entropy can be called uncertainty, meaning that it is a measure of the randomness of random variables [36]. That is, the more uncertain (larger entropy) the dataset appears under a specific attribute subset, the less important this attribute subset should be. Meanwhile, from a glance of Figure 3, we can easily sort the patterns of scatter plots in terms of their significance (Figure 4). Considering the fact that a significant pattern of image always implies a small entropy, we infer that attribute subset {*a*, *b*} is the most important one, and {*c*, *d*} is the most unimportance one, while the relative importance of {*b*, *c*} lies between them. This order is consistent with that illustrated in Figure 4.

If we denote the merit of an attribute subset *S* as *Merit_S_*, then from the above, we conclude that the order of merits can be expressed as:

(5) $${\text{Merit}}_{\left\{a,b\right\}}>{\text{Merit}}_{\left\{b,c\right\}}>{\text{Merit}}_{\left\{c,d\right\}}$$

**Figure 5.**
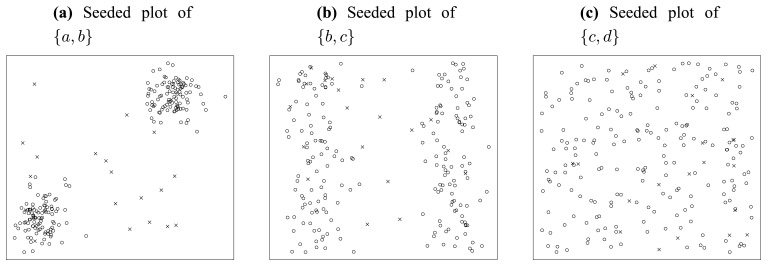
The effect of seeding. Circles are original points and crosses are the artificially injected noise points.

Next, we consider what will happen if we inject some artificial noise points into the dataset. Figure 5 shows the consequence of noise injection, where 20 uniformly distributed random points are seeded into the original dataset.

First, let us inspect the plot of attribute subset {*a*, *b*} in Figure 5(a). In this figure, we can find very clear borders between the original points marked as circles and the seeded points marked as crosses. Besides that, there are only three crosses populating in the domain of the two original normally distributed clusters. In summary, in the plot of {*a*, *b*}, the original points and the seeded points are quite distinct from each other.

Similarly, let us inspect Figure 5(b). We can find much blurred borders between the original points and the seeded points, and there are about 11 crosses populating in the domain of original points. So, in the plot of {*b*, *c*}, the original points and the seeded points are not as well separated as in Figure 5(a).

Finally, we inspect Figure 5(c). In this figure, there is no border at all. All seeded points are merged in the “ocean” of original points. It is really difficult to distinguish the seeded points from original points, without extra information provided. That is to say, the lowest significance of seeded points appears in attribute subset {*c*, *d*}, as Figure 5(c) illustrates.

As can be seen, the above 3 subplots (Figure 4(a–c)) are ordered in Figure 5, according to their significance of seeded points. Noticing that this order is consistent with that in Figure 4, we infer that the significance of artificially injected noise points is positively correlated with the merit of attributes subset. Mathematically, we denote the significance of seeded points in attribute subset *S* as *Sig_S_*, then we get:

(6) $$Sig_{\left\{a,b\right\}}>Sig_{\left\{b,c\right\}}>Sig_{\left\{c,d\right\}}.$$

Noticing that Equation (6) is consistent with Equation (5), we induce:

(7) $${\text{Merit}}_{S}\propto Sig_{S}$$

In practice, if seeded points are more significant, then they are more likely to be identified from original points. That is to say, we can evaluate the relative importance of different attribute subsets in terms of how precisely the seeded points can be detected under these attribute subsets. This is indeed what Theorem 6 (of Section 3.5) will try to tell us. Hence, through this example, we have tasted the flavour of Theorem 6 from a practical point of view.

With the above intuitions, as a starting point of the theoretical analysis, we will present the modeling of standard clustering problems in the next section.

### 3.2. Modeling of Standard Clustering

We consider a dataset *D* with *n* instances and *p* attributes (features). We can denote this dataset as an *n* × *p* matrix D. Furthermore, to denote one attribute, we express the *l*th column of D as vector *d_l_*. Besides, the *j*th data point (observation) is denoted as vector o*_j_*, which is the *j*th row of D.

Now, let us consider the standard clustering problem. If we denote the set of all possible clustering patterns as *C*, then a concrete clustering pattern can be expressed as vector c, where c ∈ *C*. First, we give the concept of clustering evaluation function.

#### Definition 1

*Clustering Evaluation Function. There is a function F* (D, c) *of data matrix D and clustering pattern* c ∈ *C. Under F, a relation R can be defined as*:

(8) $$R=\{({\text{c}}_{1},{\text{c}}_{2})|F(\text{D},{\text{c}}_{1})\geq F(\text{D},{\text{c}}_{2})\;\text{and}\;{\text{c}}_{1},{\text{c}}_{2}\in C\}.$$

*If*∀a, b, c ∈ *C the followings hold simultaneously*:

1. (a, a) ∈ *R (reflexivity);*
2. *If* (a, b) ∈ *R and* (b, c) ∈ *R, then* (a, c) ∈ *R (transitivity);*
3. *Either* (a, b) ∈ *R or* (b, a) ∈ *R (totality)*,

*then we call this function F a clustering evaluation function (CEF)*.

Essentially speaking, the relation *R* defined above can be interpreted in the sense of common “better than” relation. If a function *F* is defined, then the corresponding *R* is determined simultaneously. As a result, all the possible clustering patterns can be evaluated and compared with each other according to the function values of *F*.

Furthermore, based on the properties enumerated in Definition 1, we can define the best clustering pattern set (BCPS) as follows:

#### Definition 2

*Best Clustering Pattern Set. Set B (B* ⊂ *C) can be called a best clustering pattern set under CEF F, if*∀x ∈ *B and* ∀c ∈ *C*, (**x, c**) ∈ *R holds, where R is defined in* Equation (8).

There is an interesting result under above definition.

#### Theorem 1

∀x, y ∈ B, where B is the BCPS under Definition 2, we have F(D, x; = F(D, y;.

##### Proof

Here, we will prove it by contradiction. First, we assume that *F* (D, x) ≠ *F* (D, y). Without losing generality, we can further assume that,

(9) F(D,x)>F(D,y).

From Definition 2, we know *B* ⊂ *C*. Because x ∈ *B*, we get x ⊂ *C*. Again, from Definition 2, we can get (y, x) ⊂ *R*, that is,

(10) F(D,x)≤F(D,y).

Because Equation (10) contradicts Equation (9), we conclude,

F(D,x)≤F(D,y).

Generally speaking, every clustering methodology has its own distinct CEF *F*, and because of the preceding discussions, the standard clustering problem can be expressed as an optimization problem.

#### Definition 3

*Standard Clustering Problem. The standard clustering problem can be defined to be an optimization problem as*

(11) $$\underset{\text{c}\in C}{\max}\{F(\text{D},\text{c})\},$$

*where F(D, c) is a CEF*.

Together with Definition 3, theorem 1 clarifies a simple truth, saying that all the clustering patterns in BCPS have equally maximized CEF value, which can be found out by solving the maximization problem expressed in Equation (11). That is to say, if and only if under cluster patterns in BCPS, the target dataset D can be clustered most effectively, in terms of a specific CEF *F*.

To make the above theories more concrete, the standard *k*-means clustering will be investigated here. Given a dataset *D* of observations (o_1_, o_2_, …, o*_n_*), where each observation is a *p*-dimensional real vector, *k*-means clustering aims to partition the *n* observations into *k* sets (*k* ≤ *n*) c = (*S*_1_, *S*_2_, …, *S_k_*) so as to minimize the within-cluster sum of squares (WCSS) [34]:

(12) $$\underset{\text{c}\in C}{\min}\left\{\sum_{i=1}^{k}\sum_{{\text{o}}_{j}\in S_{i}}{\left\|{\text{o}}_{j}-\mu_{i}\right\|}^{2}\right\},$$

where *μ_i_* is the mean of points in *S_i_*, and *C* is the set of all possible clustering patterns. The minimization problem in Equation (12) can also be expressed as the following maximization problem:

(13) $$\underset{\text{c}\in C}{\max}\left\{-\sum_{i=1}^{k}\sum_{{\text{o}}_{j}\in S_{i}}{\left\|{\text{o}}_{j}-\mu_{i}\right\|}^{2}\right\}.$$

Thus, if we define a function as

(14) $$F_{\text{kmeans}}=-\sum_{i=1}^{k}\sum_{{\text{o}}_{j}\in S_{i}}{\left\|{\text{o}}_{j}-\mu_{i}\right\|}^{2},$$

then the optimization problem stated in Equation (13) is consistent with that in Equation (11). Next, we will prove that, the function *F_kmeans_* defined in Equation (14) is indeed a CEF for *k*-means clustering.

#### Theorem 2

Equation (14) *defines a CEF*.

Proof

According to Equation (14), for arbitrary a, b, c ∈ *C*, we have:

1. Because *F_kmeans_* (D, a) = *F_kmeans_* (D, a), we have (a, a) ∈ *R*;
2. If (a, b) ∈ *R* and (b, c) ∈ *R*, then *F_kmeans_* (D, a) ≥ *F_kmeans_* (D, b) and *F_kmeans_* (D, b) ≥ *F_kmeans_* (D, c), as a result, *F_kmeans_* (D, a) ≥ *F_kmeans_* (D, c), that is (a, c) ∈ *R*;
3. Because either *F_kmeans_* (D, a) ≥ *F_kmeans_* (D, b) or *F_kmeans_* (D, b) ≥ *F_kmeans_* (D, a) holds, then either (a, b) ∈ *R* or (b, a) ∈ *R* holds.

Thus, we know Equation (14) defines a CEF.

Theorem 2 tells us that,

(15) $$F=-\sum_{i=1}^{k}\sum_{{\text{o}}_{j}\in S_{i}}{\left\|{\text{o}}_{j}-\mu_{i}\right\|}^{2},$$

in *k*-means clustering. And the *k*-means clustering problem conforms with the definition of standard clustering problem (Definition 3).

### 3.3. Modeling of Feature-Selected Clustering

In this subsection, we will investigate a special kind of CEF, called feature-additive CEF.

#### Definition 4

Feature-additive CEF If a CEF F can be expressed as:

(16) $$F(\text{D},\text{c})=\sum_{l=1}^{p}f_{l}({\text{d}}_{l},\text{c}),$$

*where d_l_is the lth column of n × p data matrix D, then this CEF F is a feature-additive CEF, and the function f_l_(d_l_, c) is the lth feature-oriented subCEF. Accordingly, clustering methods based on this kind of CEF can be called feature-additive clustering methods*.

Hence, by substituting Equation (16) into Equation (11), we can express a feature-additive standard clustering problem as the following optimization problem:

(17) $$\underset{\text{c}\in C}{\max}\left\{\sum_{l=1}^{p}f_{l}({\text{d}}_{l},\text{c})\right\}.$$

Again, we resort to *k*-means clustering to make it more concrete.

#### Theorem 3

*K-means clustering is feature-additive*.

##### Proof

From Equation (15), we get:

(18) $$\begin{array}{c} F=-\sum_{i=1}^{k}\sum_{{\text{o}}_{j}\in S_{i}}{\left\|{\text{o}}_{j}-\mu_{i}\right\|}^{2}\\ =\sum_{i=1}^{k}\sum_{{\text{o}}_{j}\in S_{i}}\sum_{l=1}^{p}{\left(o_{jl}-\mu_{il}\right)}^{2} \end{array}$$

(19) $$=\sum_{l=1}^{p}\;[-\sum_{i=1}^{k}\sum_{{\text{o}}_{j}\in S_{i}}{\left({\text{o}}_{jl}-\mu_{il}\right)}^{2}].$$

The *o_jl_* and *μ_il_* in Equation (18) are the *l*th components of vector o*_j_* and *μ_i_* respectively. With respect to Equation (19), if we define,

(20) $$f_{l}=-\sum_{i=1}^{k}\sum_{{\text{o}}_{j}\in S_{i}}{\left({\text{o}}_{jl}-\mu_{il}\right)}^{2},$$

then from Equation (19), we have,

(21) $$F=\sum_{l=1}^{p}f_{l}.$$

Noticing *o_jl_* = *d_lj_*, we can get,

(22) $$f_{l}=-\sum_{i=1}^{k}\sum_{{\text{o}}_{j}\in S_{i}}{\left({\text{d}}_{lj}-\mu_{il}\right)}^{2}.$$

In Equation (22) *f_l_* is a function of feature vector d*_l_* and clustering pattern vector c. According to Definition 4 and Equation (21), we conclude that *k*-means clustering is feature-additive, and its feature-oriented subCEF is defined in Equation (22).

The introduction of feature-additive clustering is valuable, in the sense that the feature selection problem can be elegantly expressed as an optimization problem.

#### Definition 5

*Feature-selected Clustering Problem. There is a feature-additive CEF F, and its feature-oriented subCEF for feature l is f_l_. Thereby all the p f_l_form a vector function* **f** = (*f*_1_, *f*_2_, …, *f_p_*). *Then a feature-selected clustering problem becomes an optimization problem defined as*:

(23) $$\begin{array}{ll} \underset{\omega ,\text{c}}{\max} & \sum_{l=1}^{p}\omega_{l}f_{l}({\text{d}}_{l},\text{c})\\ \text{subject to} & \omega_{l}\in \{0,1\},1\leq l\leq p,\\ & \text{c}\in C. \end{array}$$

*Or, in the vectorial form as*:

(24) $$\begin{array}{ll} \underset{\omega ,\text{c}}{\max} & \omega \cdot \text{f}(\text{D},\text{c})\\ \text{subject to} & \omega \in \{(\omega_{1},\omega_{2},\ldots ,\omega_{p})ȣ\omega_{l}\in \{0,1\},1\leq l\leq p\},\\ & \text{c}\in C. \end{array}$$

In Equation 23, when *ω* = (1, 1, …, 1), we see that the feature-selected clustering problem can be transformed into a standard clustering problem defined in Equation (17). That is to say, the standard clustering problem is just a special case of feature-selected clustering problem, where all the features are selected. To be concrete, according to what Definition 5 suggests, we can generalize the standard k-means into a feature-selected one. Recalling the example in Figure 2, where we have given the clustering results of standard and feature-selected *k*-means respectively, we see that feature selection process is essential to *k*-means clustering, even in the case dealing with such a simple dataset.

One may wonder how the optimization problem in Equation 23 can be solved. In Equation 23, if a clustering pattern c is given, then *f_l_* (d*_l_*, c) will be determined simultaneously, as a result, the problem in Equation 23 can be treated as a standard binary integer programming (BIP) problem, which has been studied thoroughly in mathematics. For instance, the Balas additive algorithm [37] is a sort of specialized branch and bound algorithm for solving standard BIPs. Similarly, if a feature pattern *ω* is given, the problem in Equation 23 can then be treated as a standard clustering problem, by considering only the features selected by *ω*. From the above discussions, we can employ a rolling manner methodology [34] to handle the whole optimization problem. That is, first we start with a particular feature pattern *ω*, such as *ω* = (1, 1, …, 1), and then under this given feature pattern, an optimized clustering pattern c can be obtained accordingly, by a standard clustering procedure. Subsequently, we fix this c, and do a Balas BIP optimization to get a new *ω*. With this new *ω*, the above procedures could be performed iteratively until *ω* and c converge. Although this kind of rolling optimization seems feasible in theory, it cannot guarantee to give the global maximum, and often gives just a local maximum. Meanwhile, considering the enormous complexity of this method, we are still motivated to develop more effective and efficient algorithms to tackle the feature-selected clustering problem.

### 3.4. Coverability and Its Properties

As discussed previously, *k*-means clustering has some valuable properties, such as the additivity of feature-oriented subCEFs, which gives us the optimization perspective to tackle feature selection problems (Equation 23). In this subsection, we will introduce the concept of coverability, which can provide us another novel perspective for feature selection.

As we know, a clustering pattern can be expressed as a vector of point sets, denoted as c = (*S*_1_, *S*_2_, …, *S_k_*), where *S_i_* represents the *i*th cluster, which is a set consisting of the *N* (*i*) data points belonging to this cluster.

Now, let us inspect cluster *S_i_*. In this cluster, there are *N* (*i*) data points indexed by the set *I_i_=* {*i*_1_, *i*_2_, *…*, *i_N_*_(_*_i_*_)_}, satisfying *S_i_* = {o*_i_*_1_, o*_i_*_2_, …, o*_iN_*_(_*_i_*_)_}. The mean (arithmetical average) of these points is denoted as *μ_i_*. That is:

(25) $$\mu_{i}=\frac{\sum_{j=1}^{N(i)}{\text{o}}_{i_{j}}}{N(i)}.$$

Then, the mean-squared error (MSE) for cluster *S_i_* is:

(26) $${\text{MSE}}_{i}=\frac{\sum_{j=1}^{N(i)}{\left\|{\text{o}}_{i_{j}}-\mu_{i}\right\|}^{2}}{N(i)}.$$

If we treat $\sqrt{{\text{MSE}}_{i}}$ as a kind of radius, then we have:

#### Definition 6

*Effective Radius and Effective Circle. Regarding cluster S_i_, we call a radius ρi satisfying* $\rho_{i}^{2}={\text{MSE}}_{i}$ *or* $\rho_{i}=\sqrt{{\text{MSE}}_{i}}$ *the effective radius of cluster S_i_. Accordingly, the circle centered at μ_i_with radius ρ_i_is the effective circle of cluster S_i_*.

**Figure 6.**
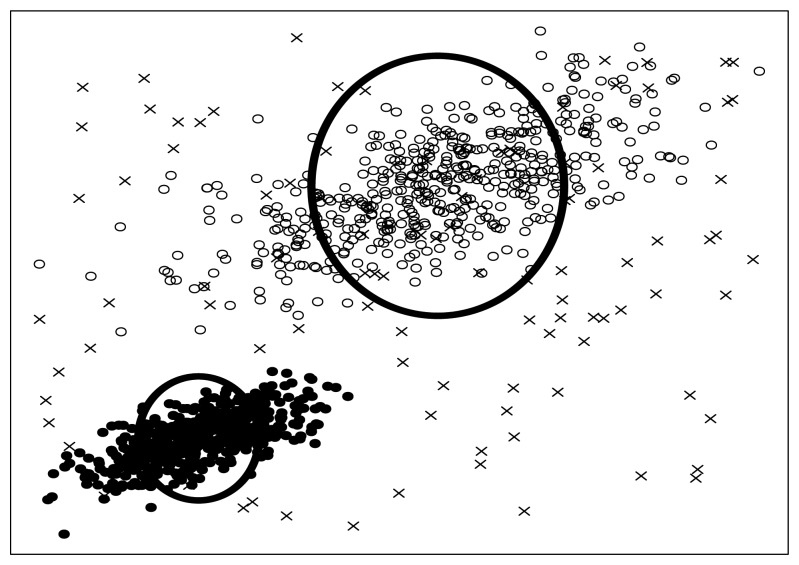
An example. Bold circles are effective circles for the two clusters respectively. Those little circles are original data points, and crosses are seeded points with uniform distribution law. This dataset has been optimally clustered, and the points belonging to the left-bottom cluster have been marked by solid circles.

As we know, $\sqrt{{\text{MSE}}_{i}}$ can be regarded as the standard deviation of samples in cluster *S_i_*. Appealing to Definition 6, the effective radius *ρ_i_* measures how widely the instances in *S_i_* are spread. Accordingly the effective circle vaguely confines the space of influence of cluster *S_i_*. To be concrete, the two bold circles in Figure 6 illustrate effective circles visibly.

With above definitions, we can give the rigorous definition of coverability now.

#### Definition 7

*Coverability. The coverability for a dataset is the infimum of the sum of N(i)-weighted* $\rho_{i}^{2}$, *where ρ_i_is the effective radius of S_i_. That is*

(27) $$ℭ=\underset{\text{c}\in C}{\text{inf}}\left\{\sum_{i=1}^{k}N(i)\cdot \rho_{i}^{2}\right\}.$$

The following theorem can help us to interpret the essence of coverability more deeply.

#### Theorem 4

*The coverability of a dataset is equal to the infimum of WCSS*.

Proof

Because $\rho_{i}^{2}={\text{MSE}}_{i}$, we have

(28) $$\begin{array}{cc} ℭ & =\underset{\text{c}\in C}{\inf}\left\{\sum_{i=1}^{k}N(i)\cdot {\text{MSE}}_{i}\right\}\\ & =\underset{\text{c}\in C}{\inf}\left\{\sum_{i=1}^{k}N(i)\cdot \frac{\sum_{j=1}^{N(i)}{\left\|{\text{o}}_{i_{j}}-\mu_{i}\right\|}^{2}}{N(i)}\right\}\\ & =\underset{\text{c}\in C}{\inf}\left\{\sum_{i=1}^{k}\sum_{j=1}^{N(i)}{\left\|{\text{o}}_{i_{j}}-\mu_{i}\right\|}^{2}\right\}. \end{array}$$

Because the infimum of WCSS for a specific dataset is definite, Theorem 4 essentially tells us that the coverability is an intrinsic property for a dataset and independent of any concrete clustering method. Reviewing Theorem 4, one may ask that, isn't WCSS good enough? And why did we bother to introduce the concept of coverability? Roughly speaking, what Theorem 4 presented is just one perspective to interpret the concept of coverability. And the essence of coverability can only be exposed from another point of view, where coverability is interpreted as the ability of a dataset to cover seeded points and make them difficult to identify. We will explain this in detail below.

What are seeded points? Look at Figure 6 again, some artificial noise points (the crosses in Figure 6) are injected into the original dataset. We call these artificial noise points seeded points or just seeds for short.

To determine the quantity of seeds, we denote the number of seeded points as *N*_0_. Hence, we can define the signal-to-noise ratio to be

(29) $$\text{SNR}=\frac{n}{N_{0}},$$

where the total number of instances in original dataset is denoted as *n* as before. In the example of Figure 6, we adopt SNR **=** 10. Besides that, we should also note that, the seeded points are uniformly distributed into the data space spanned by the original data points. We will discuss the SNR and distribution law again in Section 3.6.

Now, let us try to interpret the term— $N(i)\cdot \rho_{i}^{2}$ of Equation (27), when the infimum has been achieved. From Figure 6, we can see that if a seeded point is totally covered by a cluster, it will be very difficult to be identified from the original points, thus we can call it a faded seed. In contrast, if a seeded point departs from any cluster far enough, then it is distinct and can be extracted easily, so we call it a distinct seed. For a specific cluster *S_i_*, recalling that the area $\rho_{i}^{2}$ (we do not care about the constant π here) of the corresponding effective circle is a measurement of the range of this cluster, we can infer that, the bigger the effective circle is, the better the coverability will be, as a result more seeds will be faded. Besides, the number of points in *S_i_* (*N* (*i*)) is another important factor that is tightly relevant to coverability. Assuming that two clusters with the same size of effective circles are given, we can easily infer that the cluster with more data points is prone to higher density, hence it is more capable of covering seeded points, and eventually will be superior in coverability. Through the above discussions, Equation (27) as a whole can be interpreted as the overall seed-covering ability of all the clusters in a dataset, when the WCSS has been minimized.

Next, let us consider the probability *P*, with which the seeded points can be distinguished from the original data points correctly. From the above analysis, it is obvious that *P* is closely related with the coverability of a dataset. If the coverability is larger, then a seeded point is more likely to be covered by a cluster less likely to be detected by an outlier detector. Thus we can infer that *P* is inversely proportional to the coverability of a dataset.

From the above, we can summarize and make our fundamental hypothesis as follows.

#### Hypothesis 1

*The probability P, with which the uniformly seeded noise points can be detected correctly, is negatively correlated with the coverability C of a dataset*.

As we have pointed out, coverability is an intrinsic property for a dataset, hence Hypothesis 1 essentially tells us that *P* is also an intrinsic property for a dataset. We can explain it in this way that if a dataset is given, then how possibly the seeded points can be detected is determined accordingly. Furthermore, if we treat the uniformly injected seeded points as outliers against the original dataset, then we can adopt a particular outlier detector to evaluate *P*. Because *P* is determined on a concrete dataset if the outlier detector is given, the validity of Hypothesis 1 only depends on the characteristic of the outlier detector we adopted. That leads to the definition of ideal outlier detector as follows.

#### Definition 8

*Ideal Outlier Detector. An outlier detector is an ideal outlier detector if and only if Hypothesis 1 holds when this outlier detector is adopted*.

Essentially speaking, the requirement that Hypothesis 1 imposes on an outlier detector is that the correct detection probability should be negatively correlated with the space covered by the original points. This requirement is so loose that Hypothesis 1 seems to be a characteristic feature of outlier detectors in general. In this paper, whenever we talk about an outlier detector, we exclusively refer to the ideal outlier detector, where Hypothesis 1 holds. In practice, the validity of Hypothesis 1 can be verified phenomenologically by experiments or mechanistically by theories. Through plenty of experiments and theoretical investigations, we have found that most existing outlier detectors can be treated as ideal outlier detectors to some extent. It again confirms that Definition 8 reveals a sort of general property for outlier detectors. In this paper, we will give a detailed description of the uniformly partitioning-based outlier detector in Section 4.1. Furthermore, in Section 4.2 we will prove that it conforms to Hypothesis 1.

### 3.5. Feature-Projected Coverability and Its Properties

From now on, we will take the feature selection effect into consideration, which is indicated by the vector *ω* as before. With feature selection, an observation o can be projected into a feature-selected vector o_|_*_ω_* defined as

(30) $${\text{o}}_{|\omega }=\omega \cdot \text{o},$$

where only the components corresponding to the “1” elements of *ω* are relevant and survived from feature selection. According to Equation (30), we have the following results in the feature-selected situation, by improving Equation (25) and Equation (26).

For cluster *S_i_*, the mean of this cluster in the feature-selected circumstances is denoted as *μ*_|_*_ω,i_*. That is

(31) $$\mu_{|\omega ,i}=\frac{\sum_{j=1}^{N(i)}{\text{o}}_{|\omega ,i_{j}}}{N(i)}.$$

Then, the feature-selected mean-squared error (MSE_|_*_ω_*) for cluster *S_i_* is

(32) $${\text{MSE}}_{|\omega ,i}=\frac{\sum_{j=1}^{N(i)}{\left\|{\text{o}}_{|\omega ,i_{j}}-\mu_{|\omega ,i}\right\|}^{2}}{N(i)}.$$

Analogously to Definition 6, we can define

(33) $$\rho_{|\omega ,i}=\sqrt{{\text{MSE}}_{|\omega ,i}}.$$

Thus, similar to Definition 7, the coverability for a feature-selected dataset can be defined as

(34) $${ℭ}_{|\omega }=\underset{\text{c}\in C}{\inf}\left\{\sum_{i=1}^{k}N(i)\cdot \rho_{|\omega ,i}^{2}\right\}.$$

With the above discussions, we can define the optimal feature pattern as follows.

#### Definition 9

*Optimal Feature Pattern. We call a feature pattern ω_o_the optimal feature pattern if*

(35) $$\omega_{o}=\underset{\omega }{\arg\;\min}\{{ℭ}_{|\omega }\},$$

*where ω*∈ {(*ω*_1_, *ω*_2_, …,*ω_p_*)*|ω_l_*∈ {0, 1}, 1 ≤ *l* ≤ *p*}.

Again, we would like to explain Definition 9 in a concrete manner by investigating *k*-means clustering. The following theorem will reveal the underlying relationship between optimal feature pattern and the optimization problem defined in Equation 23.

#### Theorem 5

*In feature-selected k-means clustering, the maximum of* Equation 23 *can be achieved if and only if the features are selected according to the optimal feature pattern ω_o_defined in Definition 9*.

##### Proof

From Equation (30), Equation (32), Equation (33), and Equation (34), we get

(36) $$\begin{array}{cc} {ℭ}_{|\omega } & =\underset{\text{c}\in C}{\inf}\left\{\sum_{i=1}^{k}N(i)\cdot {\text{MSE}}_{|\omega ,i}\right\}\\ & =\underset{\text{c}\in C}{\inf}\left\{\sum_{i=1}^{k}\sum_{j=1}^{N(i)}{\left\|{\text{o}}_{|\omega ,i_{j}}-\mu_{|\omega ,i}\right\|}^{2}\right\}\\ & =\underset{\text{c}\in C}{\inf}\left\{\sum_{i=1}^{k}\sum_{{\text{o}}_{j}\in S_{i}}{\left\|\omega \cdot {\text{o}}_{j}-\omega \cdot \mu_{i}\right\|}^{2}\right\}. \end{array}$$

Next, from Equation (20) and Equation (23), we get

(37) $$\begin{array}{cc} \sum_{l=1}^{P}\omega_{l}f_{l}({\text{d}}_{l},\text{c}) & =-\sum_{i=1}^{p}\sum_{i=1}^{k}\sum_{{\text{o}}_{j}\in S_{i}}\omega_{l}{\left({\text{o}}_{jl}-\mu_{il}\right)}^{2}\\ & =-\sum_{i=1}^{k}\sum_{{\text{o}}_{j}\in S_{i}}\sum_{l=1}^{p}\omega_{l}{\left({\text{o}}_{jl}-\mu_{il}\right)}^{2}\\ & =-\sum_{i=1}^{k}\sum_{{\text{o}}_{j}\in S_{i}}\sum_{l=1}^{p}{\left(\omega_{l}{\text{o}}_{jl}-\omega_{l}\mu_{il}\right)}^{2} \end{array}$$

(38) $$=-\sum_{i=1}^{k}\sum_{{\text{o}}_{j}\in S_{i}}{\left\|\omega \cdot {\text{o}}_{j}-\omega \cdot \mu_{i}\right\|}^{2}$$

The reason for Equation (37) is $\omega_{l}=\omega_{l}^{2}$, because *ω_l_*∈ {0, 1}.

By comparing Equation (38) with Equation (36), we know that $\sum_{l=1}^{p}\omega_{l}f_{l}({\text{d}}_{l},\text{c})$ will be maximized if and only if C_|_*_ω_* is minimized. Hence the theorem is verified.

Essentially speaking, Theorem 5 reveals an important fact that, the feature selection task for *k*-means clustering can be accomplished by finding the feature pattern under which the smallest coverability is achieved. Furthermore, one may wonder whether we could find a simpler methodology to evaluate coverability instead of solving the optimization problem in Equation (35). Fortunately, Hypothesis 1 offers us a great source of inspiration. From Hypothesis 1, we know that the coverability of a dataset is coupled with the probability *P* with which the seeded points can be detected correctly. Similarly, in the feature-selected situation, we may also expect to evaluate the coverability C_|_*_ω_* by assessing the probability with which the seeded points can be correctly identified from the dataset under feature pattern *ω*. With this novel methodology, we could easily compare the coverabilities under various feature patterns to get the best one, which is potentially an answer to the feature selection problem.

To make above discussions rigorous, first of all, we give a corollary of Hypothesis 1.

#### Corollary 1

*The probability P_|ω_with which the uniformly seeded noise points can be correctly detected under a particular feature pattern ω is negatively correlated with the coverability C_|ω_under this feature pattern ω*.

Corollary 1 is straightforward. If we treat the feature-selected database as a new database, then in this new database, *P*_|_*_ω_* can be viewed as a new *P* and C_|_*_ω_* can be viewed as a new C. Via Hypothesis 1, we can easily verify what Corollary 1 stated. By Corollary 1, we get the fundamental theorem below.

#### Theorem 6

*The maximum of P_|ω_can be achieved if and only if the features are selected according to the optimal feature pattern ω_o_defined in Definition 9. Or equivalently*,

(39) $$\omega_{o}=\underset{\omega }{\arg\;\max}\{P_{|\omega }\}.$$

##### Proof

Because of Equation (35) and Corollary 1, the statement of this theorem holds obviously.

Theorem 6 tells us that we can accomplish feature selection tasks by finding the particular feature pattern under which the seeded points can be extracted most probably. This methodology is simpler and more feasible than solving the optimization problem in Equation 23. To clarify the validity of this methodology, first let us consider the *k*-means clustering. According to Theorem 5, we know that, for *k*-means clustering, the optimal feature pattern that Theorem 6 provides us is actually the solution to the optimization problem expressed in Equation 23. Then, how about a common situation? As we know, coverability is virtually the minimized WCSS of a dataset. So Theorem 6 actually gives us a practical methodology to find the feature pattern under which WCSS can be minimized. This interpretation reveals that, essentially, Theorem 6 is consistent with existing feature selection criteria [15] in the sense of minimizing WCSS. Hence, Theorem 6 is sensible in a common sense.

### 3.6. Remaining Problems

There are still some remaining problems, which need to be discussed in detail.

**How can we determine a suitable SNR?** As stated previously, SNR **=** 10 has been adopted in the example illustrated in Figure 6. To explain this, we should note that the quantity of seeded points cannot be too large. Otherwise, the seeded points will overwhelm the whole data space, and then the distinguishability of feature patterns will suffer. Meanwhile, there should not be too few seeded points either. Otherwise, the granularity becomes so coarse that it will dramatically degrade the precision of feature subset evaluation. Finally, through a lot of experiments, we found that, *P*_|_*_ω_* in Equation (39) is substantially insensitive to SNR when SNR is set moderately, and we see that SNR = 10 is a good choice in practice.

**Why did we adopt the uniform distribution for seeding?** As stated previously, coverability can be viewed as the ability for a dataset to occupy the data space in which the seeded points are spread. The number of the seeded points that have been affected by the original dataset can be used to assess the space occupation of the original dataset only when the seeded points are spread uniformly. Thus, uniform distribution is the only sensible choice.

## 4. Practical Considerations

In this section, we are mainly planning to explain two important components of our framework in detail, namely the harvester and the searcher. Next, let us talk about our uniformly partitioning-based harvester as a beginning.

### 4.1. Uniformly Partitioning-Based Harvest Method

As stated above, if we treat the seeded points as outliers in original data points, the harvest procedure is essentially an outlier detection process. There are a lot of state-of-the-art methods that can be employed. In this paper, a recent uniformly partitioning-based method called ordinal isolation [16] is adopted because it has some substantial advantages as follows:

- It is simple and fast, with *O* (*n*) complexity.
- It is scalable, because it arranges its main computations in a tree, whose branches can be pruned out during the proceeding of the whole algorithm.

More details for this algorithm can be found in the literature [16].

In this paper, although we adjusted the ordinal isolation algorithm somehow to be more suitable for our harvest tasks, we do not want to repeat the main principles of ordinal isolation here, which can be found thoroughly in the literature. However, we will try to present the detailed processing procedures of the harvester in a more practical way. That is, we will consider the simple example given in Section 3.1 again, and show the detailed processing procedures of harvester towards this simple problem.

**Figure 7.**
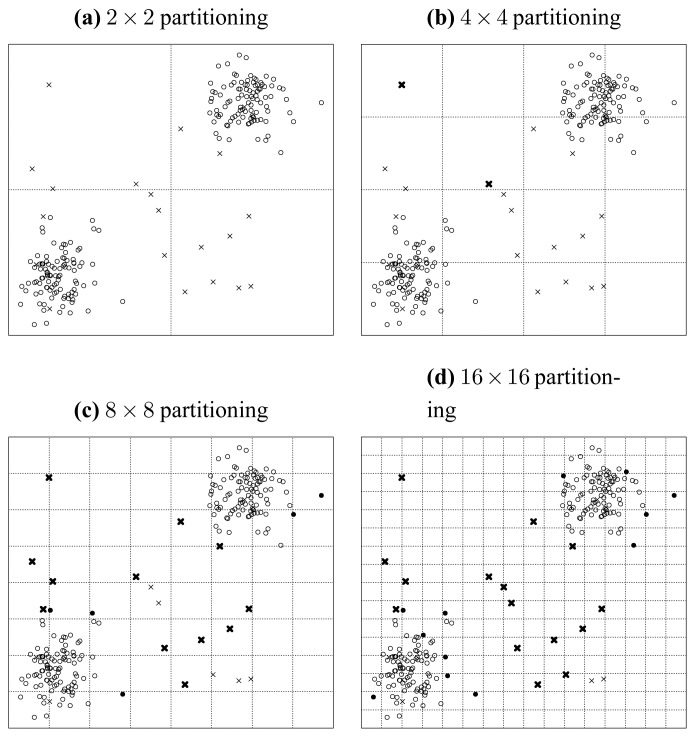
Recursively and uniformly partitioning on attribute subset {*a*, *b*}.

**Figure 8.**
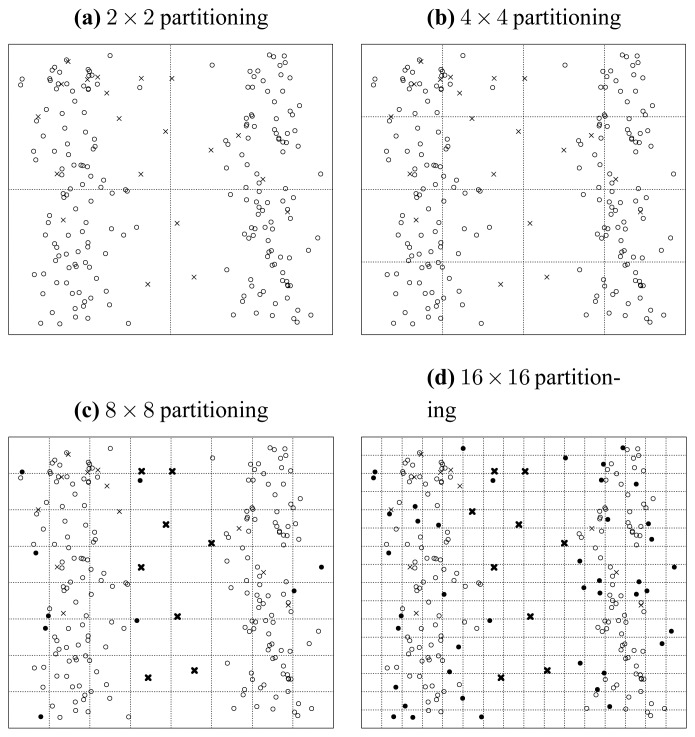
Recursively and uniformly partitioning on attribute subset {*b*, *c*}.

**Figure 9.**
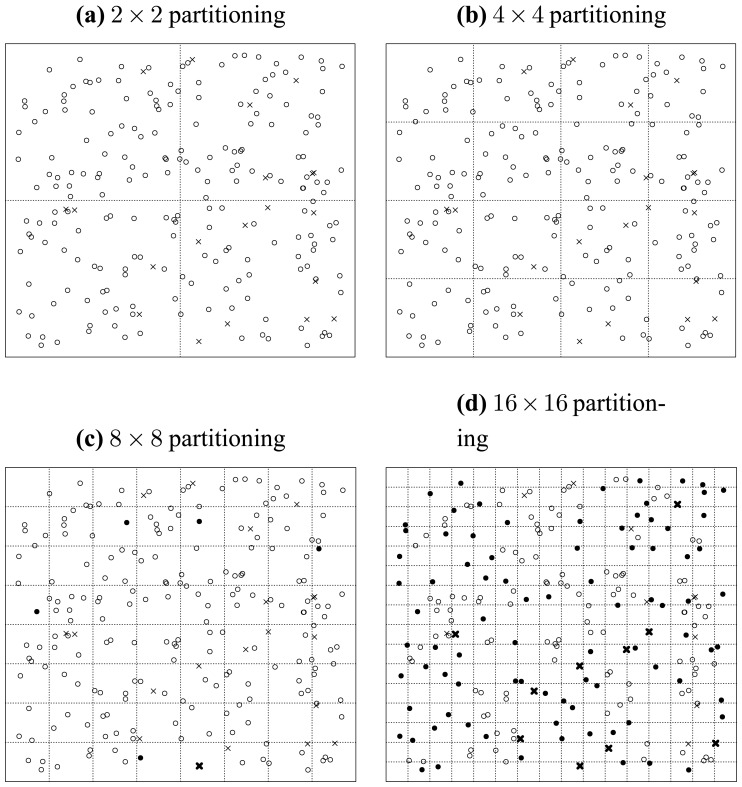
Recursively and uniformly partitioning on attribute subset {*c*, *d*}.

Figure 7, Figure 8 and Figure 9 illustrate the recursively and uniformly partitioning processes on attribute subsets {*a*, *b*}, {*b*, *c*} and {*c*, *d*} respectively. The first subplots of each above figures show the initial 2 × 2 uniformly partitioning, which split each attribute uniformly into two equal halves. Then, we get the remaining subplots by carrying out the same uniform partitioning operation recursively, which generates the 4 × 4, 8 × 8 and 16 × 16 partitioning schemes sequentially. In each figure, the seeded points are marked as crosses, and original points are marked as circles. If a seeded point is isolated, we denote it as a dark cross. Similarly, we mark isolated original points as dark disks.

We denote the operation of counting the number of isolated seeded points (dark crosses) as S(*S*, *l*), where *S* is the attribute subset and *l* represents a 2*^l^* × 2*^l^* partitioning. Similarly, we denote the operation of getting the number of isolated original points (dark disks) as O(*S*, *l*). Then from Figure 7, we can count the numbers of isolated points, and get:

(40) $$\begin{array}{ccc} \{\begin{array}{l} S(\{a,b\},1)=0\\ S(\{a,b\},2)=2\\ S(\{a,b\},3)=12\\ S(\{a,b\},4)=15 \end{array} & \text{and} & \{\begin{array}{l} O(\{a,b\},1)=0\\ O(\{a,b\},2)=0\\ O(\{a,b\},3)=5\\ O(\{a,b\},4)=2 \end{array} \end{array}.$$

If we define *Merit_S,l_* (where *S* and *l* have the same meanings as those in S(*S*, *l*)) as the following:

(41) $${\text{Merit}}_{S,l}=\frac{S(S,l)}{O(S,l)},S(S,l)>0\;\text{and}\;O(S,l)>0,$$

then we get the following equations:

(42) $$\{\begin{array}{c} {\text{Merit}}_{\{a,b\},3}=12/5=2.40\\ {\text{Merit}}_{\{a,b\},4}=15/12=1.25 \end{array},$$

by appealing to Equation (41).

Analogously, from Figure 8, we get:

(43) $$\begin{array}{ccc} \{\begin{array}{l} S(\{b,c\},1)=0\\ S(\{b,c\},2)=0\\ S(\{b,c\},3)=8\\ S(\{b,c\},4)=9 \end{array} & \text{and} & \{\begin{array}{l} O(\{b,c\},1)=0\\ O(\{b,c\},2)=0\\ O(\{b,c\},3)=9\\ O(\{b,c\},4)=43 \end{array} \end{array}.$$

Appealing to Equation (41), we get:

(44) $$\{\begin{array}{c} {\text{Merit}}_{\{b,c\},3}=8/9=0.89\\ {\text{Merit}}_{\{b,c\},4}=9/43=0.21 \end{array}.$$

Finally, from Figure 9, we get:

(45) $$\begin{array}{ccc} \{\begin{array}{l} S(\{c,d\},1)=0\\ S(\{c,d\},2)=0\\ S(\{c,d\},3)=1\\ S(\{c,d\},4)=10 \end{array} & \text{and} & \{\begin{array}{l} O(\{c,d\},1)=0\\ O(\{c,d\},2)=0\\ O(\{c,d\},3)=5\\ O(\{c,d\},4)=85 \end{array} \end{array}$$

Appealing to Equation (41), we get:

(46) $$\{\begin{array}{c} {\text{Merit}}_{\{c,d\},3}=1/5=0.20\\ {\text{Merit}}_{\{c,d\},4}=10/85=0.12 \end{array}.$$

Thus, from Equation (42), Equation (44) and Equation (46), we get:

(47) $$\{\begin{array}{c} {\text{Merit}}_{\{a,b\},3}>{\text{Merit}}_{\{b,c\},3}>{\text{Merit}}_{\{c,d\},3}\\ {\text{Merit}}_{\{a,b\},4}>{\text{Merit}}_{\{b,c\},4}>{\text{Merit}}_{\{c,d\},4} \end{array}.$$

Note that the order given in Equation 47 is consistent with that given in Equation (5) and Figure 4. So we can induce that *Merit_S,l_* can be treated as a merit order indicator for attribute subsets, by which the order but not exact values of the merits of different attribute subsets can be preserved, as Equation 47 and Equation (5) exhibit. In the next subsection, we will address why this uniformly partitioning-based methodology conforms to Hypothesis 1.

### 4.2. The Ideality of Uniformly Partitioning-Based Outlier Detector

As what Definition 8 reveals, the uniformly partitioning-based outlier detector can be classified as the ideal outlier detector if and only if ∀*D*, where *D* is a dataset, the possibility *P* with which the uniformly seeded noise points can be detected correctly is negatively correlated with the coverability C of a dataset. In this section, we will explain the ideality of the uniformly partitioning-based outlier detector in a more rational and rigorous way.

**Figure 10.**
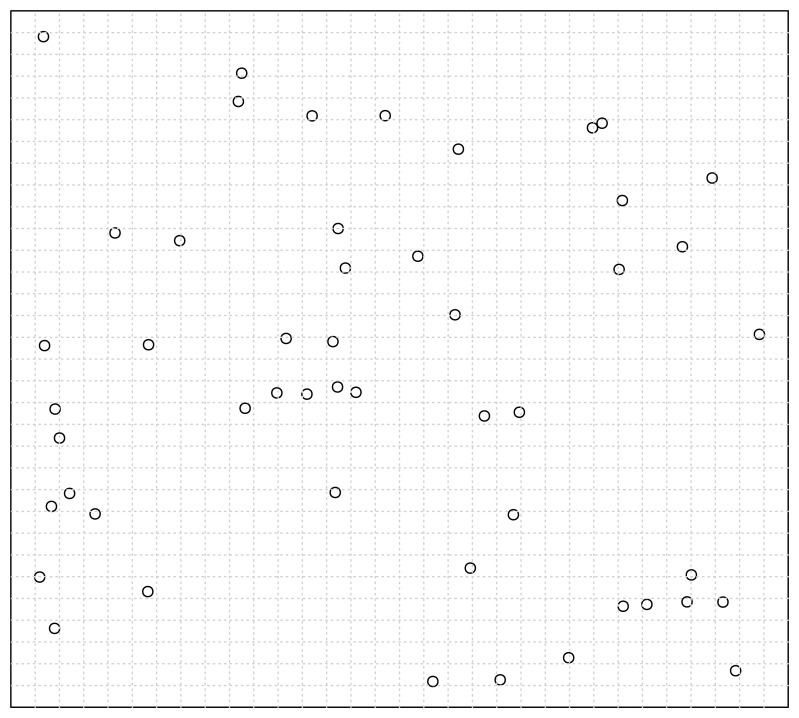
The seeded points have all been isolated in this 32 × 32 partitioning.

First, let us assume a situation illustrated in Figure 10.

In this situation, we only consider the seeded points, which are uniformly distributed in the data space. We carry out a recursively and uniformly partitioning procedure. When we reach the 32 × 32 partitioning stage, we notice from Figure 10 that all the seeded points have been isolated. Then in this situation, the ratio of correctly detected seeds can be rationally inferred to be 100%.

**Figure 11.**
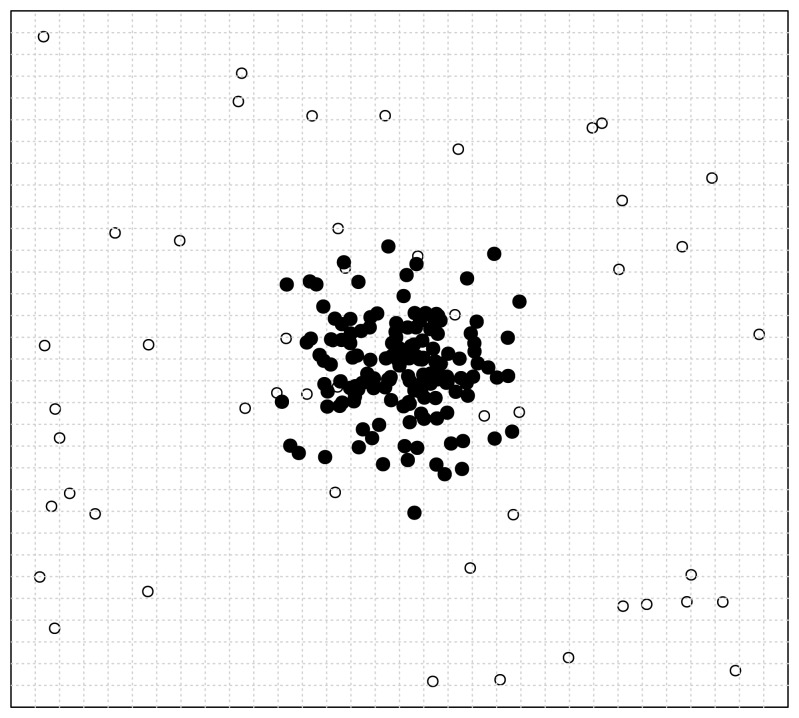
The situation when original points (solid ones) have been injected.

Then, we consider what will happen when the original data points are populated into this data space. We illustrate this situation in Figure 11, where the original points are assume to be normally distributed and indicated by solid discs. First, we investigate the case of one particular seeded point. It is obvious that when an original point locates in a cell in which a seeded point has already been located, then the distinctness of this seeded point is affected by this original point as illustrated by Figure 11.

Second, when we consider the original data points as a whole, we can see that in the middle of Figure 11 the seeded points have been covered by the original points, which consequently makes them less probable to be detected correctly. Thus, the ratio of correctly detected seeds can be rationally inferred to be much less than 100%. That is to say, the existence of original points reduces the ratio of correctly detected seeded points.

Now, let us consider how the original points act on the correct detection ratio.

First, we consider the position of the original points as a whole. That is to say, we consider the effect of a common position transposition for all the original points. In this situation, we can imagine that, because the seeded points are distributed uniformly, the state of interfering is also uniformly spread in the data space. That is to say, the transposition of original data points cannot significantly alter the correct detection ratio.

Second, we consider how the size of the original data points affects the correct detection ratio when the concentration sustains at a fixed level. As Figure 11 illustrates, the ratio of affected seeded points are positively correlated with the size of original data points. Because the concentration is fixed, we can infer that the ratio of affected points will increase with positively ascending size of original points. But the intensity of this kind of affectation will not change because of the constant concentration. As a whole, the correct detection ratio is negatively correlated with the size of original data points when the concentration is fixed.

Last, we should consider how the concentration of the original data points affects the correct detection ratio when its size sustains at a fixed level. In this situation, it is straightforwardly to see that when the ratio of affected points is fixed, if the concentration is increased, then it will be more likely that the original points can be isolated, which results in the detection of the original points rather than the seeded points and thus reduces the ratio of correct detection. So, as a whole, the correct detection ratio is negatively correlated with the concentration of original data points when its size is fixed.

Until now, we have been armed enough to investigate how the coverability of original points is correlated with its size and concentration. As we have discussed, the coverability of a dataset depict its space-covering ability. And, as we proved in Theorem 4, the coverability of a dataset is equal to the infimum of WCSS. We can conclude that the coverability of original points is positively correlated with its concentration and size.

Generally speaking, from the above discussions, we can conclude that the coverability of original points is negatively correlated with the possibility (ratio) of correct detection. That is to say, the uniformly partitioning-based outlier detector we adopted is indeed one particular type of ideal outlier detectors.

In the next subsection, we will address why the “order” is superior to the “value” and explain the main principles of ordinal searching methodologies.

### 4.3. Ordinal Searching Principle

**Figure 12.**
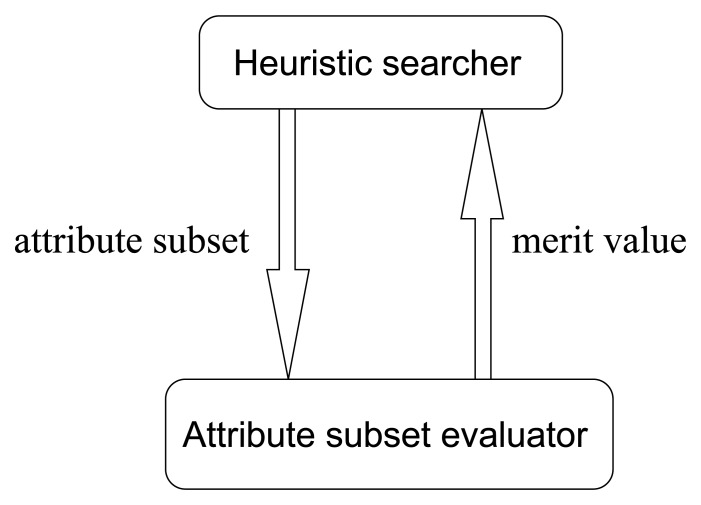
Schema of traditional value-based feature selection.

Most traditional heuristic searching methodologies are value-based, where the searching directions are determined according to the merit values of attribute subsets. The cooperating pattern between heuristic searchers and attribute subset evaluators is illustrated in Figure 12.

From Figure 12, it is obvious that in traditional value-based searchers, there are a lot of merit values that need to be evaluated in each step of searching. To be concrete, let us consider the greedy hill climbing method, which is a simple but common kind of searcher. In one step of greedy hill climbing, the attribute with the highest merit gain is added into the attribute subset, which will be treated as the searching result when the merit value cannot be further enhanced by adding any individual attribute. Hence, the essential operation in one step is evaluating a sequence of attribute subsets and fetching the one with the best merit. As we know, in high-dimensional circumstances, considering the potential huge number of merit values to evaluate, we see that this value-based manner is really time-consuming. Then, one may ask, if what we want to find out is just the best one, why do we bother to evaluate all the merit values? Can we abandon the concern with concrete merit values, and just produce a descendingly ordered sequence of attribute subset somehow, and then pick the first one? Is the order more feasible than the value? Is the ordinal searching methodology better?

The above questions are straightforward to answer. Let us take an example. If Tom is 1.75*m* tall, and Jack is 1.88*m* tall, then the conclusion “Jack is taller than Tom” will be much easier to get than the conclusion “Jack is 0.13*m* taller than Tom”. This argument is elaborated by the two well-known principles [38] in ordinal optimization theory:

1. “Order” is much more robust against noise and easier than “Value”.
2. Do not insist on getting the “Best” but be willing to settle for the “Good Enough”.

**Figure 13.**
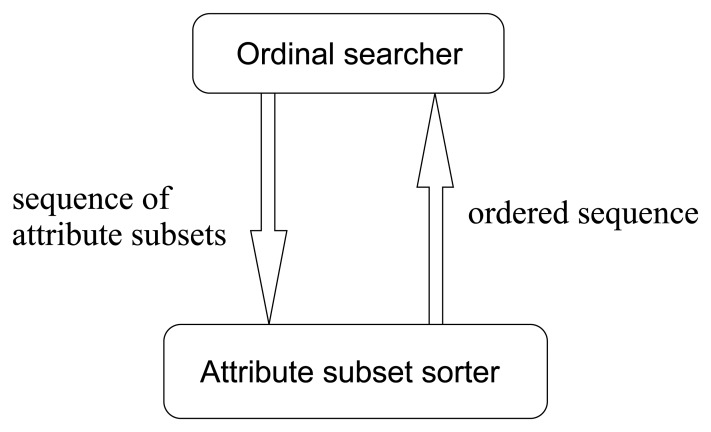
Schema of novel order-based feature selection.

So, in this paper, we improve the traditional value-based search methods into order-based ones. Accordingly, the value-based pattern in Figure 12 turns into the ordinal pattern illustrated in Figure 13. This is a novel searching methodology in avoiding the evaluations of merit values, by means of merit order indicators such as *Merit_S,l_* defined ascendingly to sort the input sequence of attribute subsets. This methodology can not only save a lot of computing time but also produce more robust results.

The last question is: how we can get the order of attribute subsets by means of our seeding and harvest framework? Appealing to Equation 47, whose order is consistent with that given in Equation (5) and Figure 4, we see that the attribute subsets have been perfectly ordered in level *l*, where the numbers of isolated seeded points and isolated original points in each attribute subset are all non-zero for the first time. For instance, the order can be determined by Equation (5) when *l* = 3, and this order will sustain when *l* > 3, so this property can be used to reduce computing complexity by pruning off the computations beyond level *l*, where ∀*S*, S(*S*, *l*) > 0 and O(*S*, *l*) > 0 hold. We will give all the implementation details in the next section.

## 5. Implementation

From previous discussions, we see that our seeding and harvest framework is capable of sorting the input attribute subsets in terms of their relative importance. This order is used by order-based searcher to determine the direction for the next searching step. The main structure of their cooperation has been illustrated in Figure 13. In this section, we will exhibit the implementation details of all the relevant algorithms. First, let us talk about the order-based searching algorithms.

### 5.1. Ordinal Searcher

In AI, heuristic search is a metaheuristic method for solving computationally hard optimization problems. Heuristic search can be used on problems that can be formulated as finding a solution maximizing a criterion among a number of candidate solutions. Heuristic search algorithms move from solution to solution in the space of candidate solutions (the search space) by applying local changes, until a solution deemed optimal is found or a time bound has elapsed [39].

There are a lot of state-of-the-art heuristic searching algorithms that can be adopted in the feature selection applications. In this subsection, we will show how the simple greedy hill climbing searching algorithm can be transformed into a corresponding order-based one.

First, Algorithm 1 gives the traditional value-based greedy hill climbing searching method.


**Algorithm 1**
*greedy_hill_climbing_search*
 1:*s* ← start state. 2:Expand *s* by making each possible local change. 3:Evaluate each child *t* of *s*. 4:*s*′ ← *t* with the highest *Merit* (*t*) 5:**if**
*Merit* (*s*′) ≥ *Merit* (*s*) **then** 6: *s* ← *s*′, **goto** 2 7:**end if** 8:**return**
*s*


In this algorithm, we evaluate all the possible directions for the next step and pick the direction with the highest merit gain. Obviously, it is value-based, because it depends on merit values and comparisons.

Then, we transform Algorithm 1 into an order-based searching algorithm, which is elaborated in Algorithm 2.


**Algorithm 2**
*ordinal_greedy_hill_climbing_search*
 1:*s* ← start state. 2:Expand *s* by making each possible local change. 3:Make a *list* consists of *s* and each child *t* of *s*. 4:*ordered*_*list* ← *attribute*_*subset*_*sorter* (*list*) 5:*h* ← *head*_*of* (*ordered*_*list*) 6:**if**
*h* ≠ *s*
**then** 7: *s* ← *h*, **goto** 2 8:**end if** 9:**return**
*s*


In this algorithm, the *head*_*of* () operator is used for extracting the head node of a list, and *attribute sub*_*set*_*sorter* (*list*) represents a procedure that sorts the input sequence of attribute subsets *list* into the output sequence *ordered*_*list* according to the relative importance of these attribute subsets. Hence, from this point of view, our seeding and harvest framework can be seen as a concrete implementation of the *attribute*_*subset*_*sorter* (*list*) procedure. The implementation details of S&H will be addressed in the next subsection.

The purpose of Algorithm 2 is self-explanatory. Note that a state in Algorithm 2 is virtually an attribute subset. Essentially speaking, line 4 of Algorithm 2 takes advantage of a so-called attribute subset sorter to order the sequence comprising the current state and all the possible child states derived from this state into an ordered sequence of attribute subsets. Hence the head of this sequence can then be treated as the next state, which is supposed to present the highest merit gain in practice. As we expect, the above procedure can be applied iteratively until the current state cannot be improved further. Then the corresponding attribute subset is the result of an ordinal feature selection task.

As we know, there are plenty of heuristic searching algorithms, such as best first search and genetic search. They can be transformed into ordinal-based ones analogously. In this paper, we adopt the method shown in Algorithm 2 as our ordinal searcher (Figure 13).

### 5.2. Seeding and Harvest Sorter Framework

**Figure 14.**
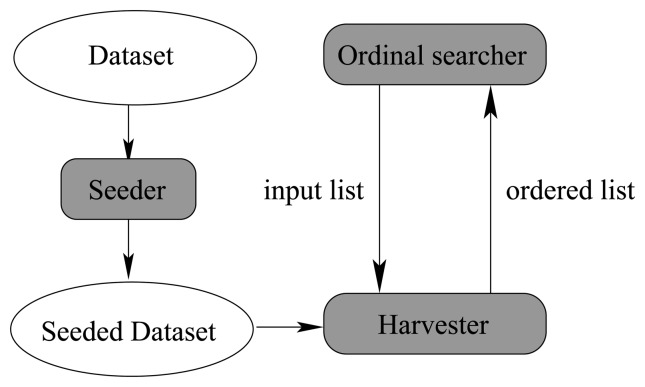
Relationship among the main components (shaded blocks).

In this subsection, we will elaborate how to sort a sequence of attribute subsets by means of our seeding and harvest framework. As discussed previously, there are three main components in our algorithm. They are the seeding component, the harvest component, and the searcher component. Figure 14 illustrates their relationship.

In Figure 14, the seeding component injects artificial noise points into the original dataset and produces the seeded dataset, which is shared among the 3 components as a global variable. The seeding component is very simple, because it is essentially a random number generator, which can produce multidimensional uniformly distributed random vectors.

The searcher component has been studied thoroughly in Algorithm 2. The harvest component is virtually an implementation of the *attribute sub*_*set*_*sorter* (*list*) procedure of Algorithm 2. It makes use of the seeded dataset and the input list to produce an ordered output list, which is fed back into the searcher component again to determine the state of next step. When the searching process cannot proceed further, the whole algorithm can stop and give the best attribute subset. Next, we will talk about the detailed algorithm of the harvest component.

**Figure 15.**
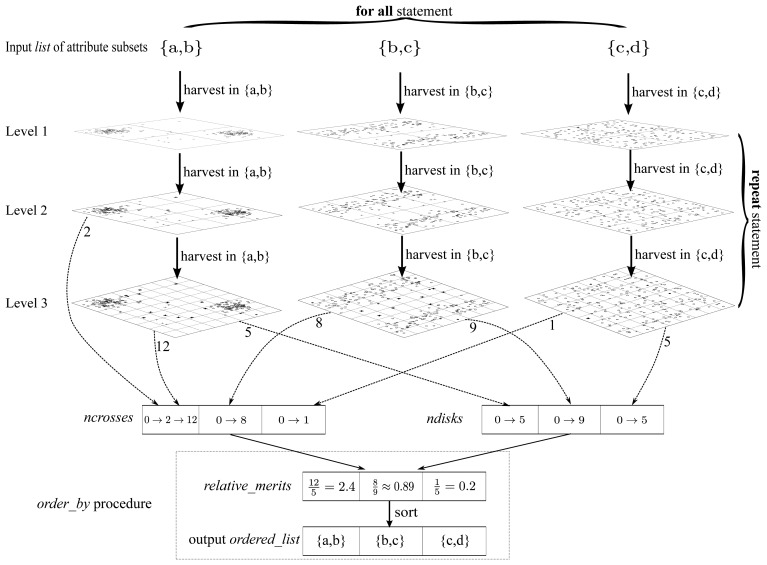
The “big” structure of harvest algorithms, where “→” means “the variable is overwritten by …”.

Algorithm 3 elaborates the detailed implementation of the harvest component. Meanwhile, to make Algorithm 3 easier to follow, we draw a really “big” graphical guidance to illustrate the main structure of Algorithm 3 in Figure 15.


**Algorithm 3**
*harvest* (*list*)
**Input:**
*list* - the list of attribute subsets to sort**Output:**
*ordered*_*list* - the output ordered list 1:initialize two arrays *ncrosses* and *ndisks* whose sizes are both |*list*|. 2:clear all the elements of *ncrosses* and *ndisks* as 0 3:**repeat** 4: **for all**
*subset* ∈ *list*
**do** 5:  *harvest*_*in*_*subset* (*subset*) 6: **end for** 7:**until** all elements in *ncrosses* and *ndisks* are non-zero 8:*ordered*_*list* ← *order*_*by* (*list, ncrosses, ndisks*) 9:**return**
*ordered*_*list*


Algorithm 3 is implemented in a “level by level” manner as illustrated in Figure 15, where the dataset is iteratively partitioned. The *harvest*_*in*_*subset* (*subset*) procedure is capable of pushing the uniformly partitioning process one level forward with respect to a particular attribute subset provided as the argument *subset* of this procedure. To be concrete, the arrows marked “harvest in {a,b}” in Figure 15 are essentially procedure calls of *harvest*_*in*_*subset* ({*a*, *b*}). Moreover, *ncrosses* and *ndisks* are two arrays of counters for bold crosses and dark disks respectively, one cell for each attribute subset. The meanings of “bold crosses” and “dark disks” are consistent with those in Figures 7–9. If a new value is produced in one level, then the corresponding counter should be updated (*i.e.*, the old value is overwritten), as operator “→” denotes in Figure 15. Besides, the *order*_*by* procedure is confined in a dotted frame as illustrated at the bottom of Figure 15. It produces the output list *ordered*_*list* according to the contents of *relative*_*merits*, which could be assessed in terms of *ncrosses* and *ndisks*. As stated previously, the *relative*_*merits* here is essentially a merit order indicator but not the true merit value. To fill *ncrosses* and *ndisks*, the “repeat” marked procedures of Figure 15, which correspond to the “repeat” statement block of Algorithm 3, proceed level by level, until all the cells in *ncrosses* and *ndisks* are non-zero. Finally, to cooperate with above iteration for levels, in each level, there is still an iteration block marked as “for all” in Figure 15, which fills contents into *ncrosses* and *ndisks* for all the attribute subsets.

Maybe there remains a dummy question. Why do we bother to give a whole ordered list as the output—can we just give the best attribute subset instead? Of course, in the greedy hill climbing search, the answer is positive, because the ordered list will be eventually used to find out the best attribute subset. However, in terms of other more sophisticated searching methodologies where more information is demanded (not just the best attribute subset) to decide the searching direction, the answer is obviously negative. The above reasoning motivates us to implement the harvest algorithm in the manner of Algorithm 3 to potentially attain more flexibility.

In the next subsection, we will analyze the complexity of our method.

### 5.3. Complexity

From Algorithm 3 we see that the whole process can stop when all the cells in *ncrosses* and *ndisks* are non-zero, which can be called the pre-pruning criterion (PPC). When PPC is satisfied, then the algorithm can be stopped. This property saves a lot of CPU-time. Through a lot of experiments, we found that the whole algorithm can complete within ℒ levels of partitioning, which is always a small constant in most circumstances, just like the example shown in Figure 15. This is an important fact, and we will take advantage of it later.

In Figure 15, there are 4 partitions for each attribute subset in level 1. This number becomes 16 in level 2. Thus, in level *l*, there are 4*^l^* partitions for each attribute subset. Therefore, the upper bound of the number of partitions for an attribute subset in each level is 4^ℒ^. Note that ℒ is a constant, so *P* = 4^ℒ^ is a constant too.

Now, let us talk about the number of attribute subsets. Here we denote the dimension of the original dataset as *d*. Appealing to Algorithm 2, if starting from the empty initial state, we know that the *list* given to harvest procedure (*attribute*_*sub*_*set*_*sorter* (*list*)) has *d* elements at the first time. In the following steps, the size of *list* is reduced to *d* − 1, *d* − 2, *d* − 3 .… Thus, the upper bound of |*list*|, which is the input size of the harvest component, is *d*.

In each level of Algorithm 3, a total scan of original dataset can achieve the partitioning mission, whose complexity is *O* (*Pnd*), where *n* is the size of dataset. So the total complexity of attribute subset sorting process is *O* (ℒ*Pnd*). Because ℒ and *P* are two constants, the complexity becomes *O* (*nd*). Because dimension *d* is much stickier than size *n*, the complexity becomes *O* (*n*) virtually, when the complexity we studied is dominated by the size of dataset.

## 6. Performance Study

In this section, we will carry out a series of experiments on plenty of real-life datasets from the UCI Machine Learning Repository [40] to evaluate several aspects of performance of our method compared with other existing methods. First, we introduce the platform we employed.

### 6.1. Platform

All experiments were conducted on an Intel Core 2 PC, with two 1.80 GHz cores, 1GB main memory. Notice that each experiment runs as a single thread, which can only be processed by one core. Our method is implemented in Java with Eclipse IDE. Our experimental platform is Weka (Waikato environment for knowledge analysis) [41], which is an excellent tool in data mining and brings together many machine learning algorithms under a common framework. Besides that, Weka is an open source software issued under the GNU General Public License, and the official website of the Weka project can be accessed at http://www.cs.waikato.ac.nz/ml/weka/. Note that the version we adopted in the following experiments is the latest version from Weka's svn system, which can be checked out from the source code repository with: *svn co* https://svn.scms.waikato.ac.nz/svn/weka/trunk/weka.

**Figure 16.**
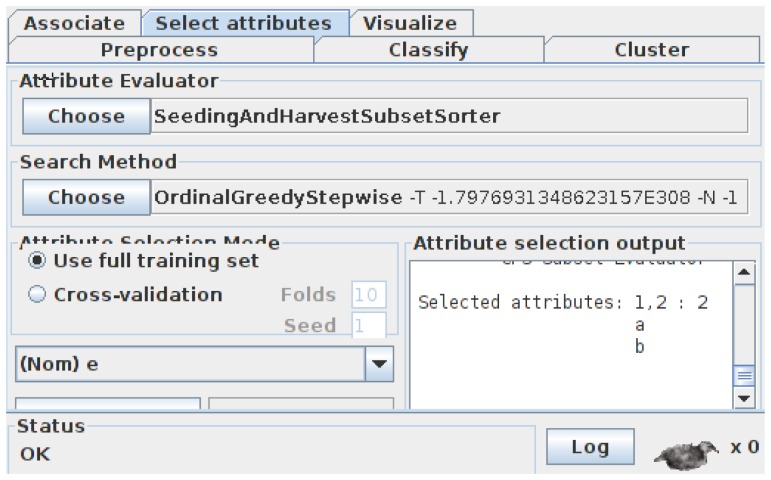
Weka Explorer using our method.

To integrate our method into Weka, first we add two main modules into the original Weka package “weka.attributeSelection”. One is “OrdinalGreedyStepwise”, which implements Algorithm 2, and the other is “SeedingAndHarvestSubsetSorter”, which implements Algorithm 3. These two modules change the traditional *subset* → *evaluate* attribute selection framework in Weka into a new *subset*_*list* → *ordered*_*list* one, for which we added the “SubsetSorter” interface into Weka. Through the above modifications, the Weka Explorer [41] can finally use our method to carry out some simple experiments. For the simple dataset illustrated in Figure 3, appealing to Figure 16, Weka gives the same result {*a*, *b*} as that given in Section 3, using our subset sorter and ordinal searcher.

**Figure 17.**
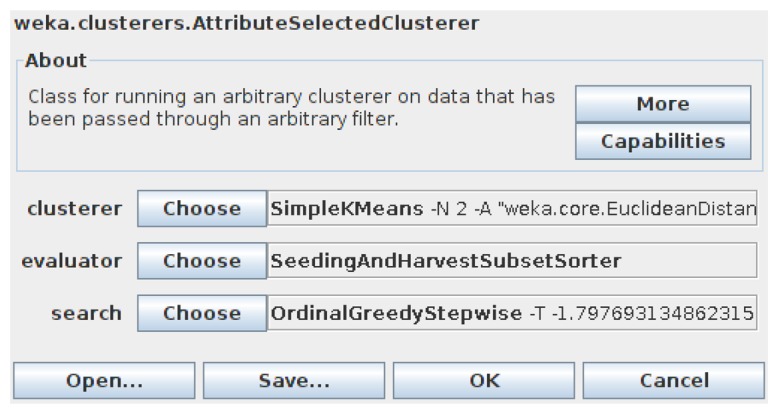
Weka Experimenter using our method.

However, this is just a beginning. In order to examine how well our method performs on given huge datasets, we must rely on the Weka Experimenter, which can do comparisons among different methods under varies conditions automatically [41]. To integrate our method into Weka Experimenter, we did the following modifications to Weka. First, we added the “AttributeSelectedClusterer” module into the “weka.clusterers” package, so that we can evaluate the contribution of attribute selection to clusterer algorithms, appealing to Figure 17.

The attribute-selected wrapper for classifiers has been implemented by Weka already, so we can use it directly. Second, for clusterers, we want to measure the squared errors to compare their performance. Therefore, we implemented “AdditionalMeasureProducer” interface for a lot of corresponding modules. Because the details are tedious, we omit them here. In the next subsection, we will introduce the datasets we used in experiments.

### 6.2. Benchmark Datasets

To rank performance evidently, we adopted 10 benchmark datasets from UCI Machine Learning Repository [40]. Their basic information is shown in Table 1.

We should notice that, although our method does not need any label (class) information since it is an unsupervised method, all the datasets we adopted contain label information, because we will compare our method with several supervised feature selection methods like CFS [5] and IG (information gain), which need classification information to evaluate merit of features or feature subsets. The number of classes of each dataset is listed in the last column of Table 1.

### 6.3. Experimental Methodology

To evaluate the performance of our method, at the beginning, we compare it with 4 classical feature reduction methods, which are CFS [5], information gain (IG), principal component analysis (PCA) [9] and relief [23,24]. Except PCA, the other three methods are all supervised ones. PCA is the only feature transformation method that transforms original features into new ones. We should also note that, except CFS and our method, all the other three methods are attribute evaluation methods, which need the attribute ranker threshold to determine how many attributes should be retained. Second, as our methodology is unsupervised, we will also carry out extra experiments to compare the performance of our method with plenty of state-of-the-art unsupervised feature selection methods, which have been reviewed briefly in Section 2. They are the FSSEM method from [29], the CEPI method from [30], the MCS method from [31], the SPECTRAL method from [32] and the SIMILARITY method from [33]. These target methods are typical and comprehensive for performance comparisons. In our experiments, we set as threshold the value that can make the best performance (least squared mean or log-likelihood) for those feature selection methods on each specific dataset. That means, we will compare the performance of our method with the best performance other methods can achieve.

How to compare the performance of feature reduction methods? As we have clarified, the main purpose of our methodology is to try to tackle the feature-selected clustering problem described in Definition 5. Hence, we employ a methodology comparing the squared errors and log-likelihoods of clusterers after feature reductions. The more significantly a feature reduction method can reduce the squared errors or increase log-likelihoods of a clusterer, the better performance this method achieves. Brief descriptions about these clusterers are given as follows.

- Standard *k*-means [41] is a simple centroid-based technique. It randomly selects *k* cluster means or centers. For each of the remaining objects, an object is assigned to the cluster to which it is the most similar, based on the distance between the object and the cluster mean. It then computes the new mean for each cluster. This process iterates until the criterion function converges. In our experiments we simply let Weka decide *k* automatically and adopt Euclidean distance.
- Hierarchical methods [41] work by grouping data objects into a tree of clusters. They can be further classified as either agglomerative or divisive, depending on whether the hierarchical decomposition is formed in a bottom-up (merging) or top-down (splitting) fashion. In our experiments, we adopt bottom-up fashion, Euclidean distance definition, and let Weka decide the number of clusters automatically.
- Simple EM (expectation maximization) methods [41] assign to each instance a probability distribution that indicates the probability of it belonging to each of the clusters. EM can decide how many clusters to create by cross validation, or one may specify a priori how many clusters to generate. Hence, there is no need to concern about the number of clusters parameter.

### 6.4. Results and Discussions

First, we present the performance comparisons with classical feature reduction methods mentioned above.

Table 2 exhibits the experimental results of standard *k*-means clusterer (SimpleKMeans module in Weka). In this table, S&H denotes our seeding and harvest method, RLF denotes relief method, UnSelect denotes the corresponding clusterer without any feature selection, and other column names are self-explanatory. Furthermore, each cell in Table 2 denotes the squared error of SimpleKMeans after carrying out a specific feature selection method (column name) on a dataset (row name). The table also shows how often each method performs significantly better (denoted as a ●) than performing no feature selection (column 2;. Throughout this paper, we speak of results being significantly different if the difference is statistically significant at the 0.05 level according to a paired two-sided *t* test.

From Table 2, we see that all feature selection methods can significantly improve the performance of SimpleKMeans, but only our method exhibits remarkable improvements on each dataset. Furthermore, if we inspect the rows of Table 2, we see that our method is always the one with the least squared error, except just two datasets, namely “magic” and “sensor”. To clarify that fact, we make our method the comparison target and summarize the results in Table 3, from which we know that our method is significantly superior (denoted as ●) to other methods in most circumstances. There is only one degradation (denoted as ○; and a few draws (blank cells;. Thus, from experimental results in Tables 2 and 3, we conclude that our method can not only significantly improve the accuracy of SimpleKMeans but also exhibit dramatic superior performance to all the other four classical feature reduction methods that we compare with.

Second, we would like to present the performance comparisons involving the five abovementioned unsupervised feature selection methods.

With the same datasets and experimental procedures of Table 3, we get Table 4.

From Table 4, we can see that our method shows superior performance than other unsupervised methods in most circumstances (32 times), and shows statically equal performances 13 times, while in the last 5 circumstances our method is worse than the target methods. Overall, from Table 4, it is enough evident to conclude that the performance of our method is generally better than the unsupervised feature selection methods that we compare with.

Next, let us inspect how fast our method can achieve. In Table 5, we make our method (S&H) the comparison target. If some method takes significant longer time than ours does, we will mark a “○” beside it. As we have known, the total run time consists of feature reduction time and clustering time. We are interested in feature reduction. Therefore, feature reduction time is put in front of total time in Table 5.

**Figure 18.**
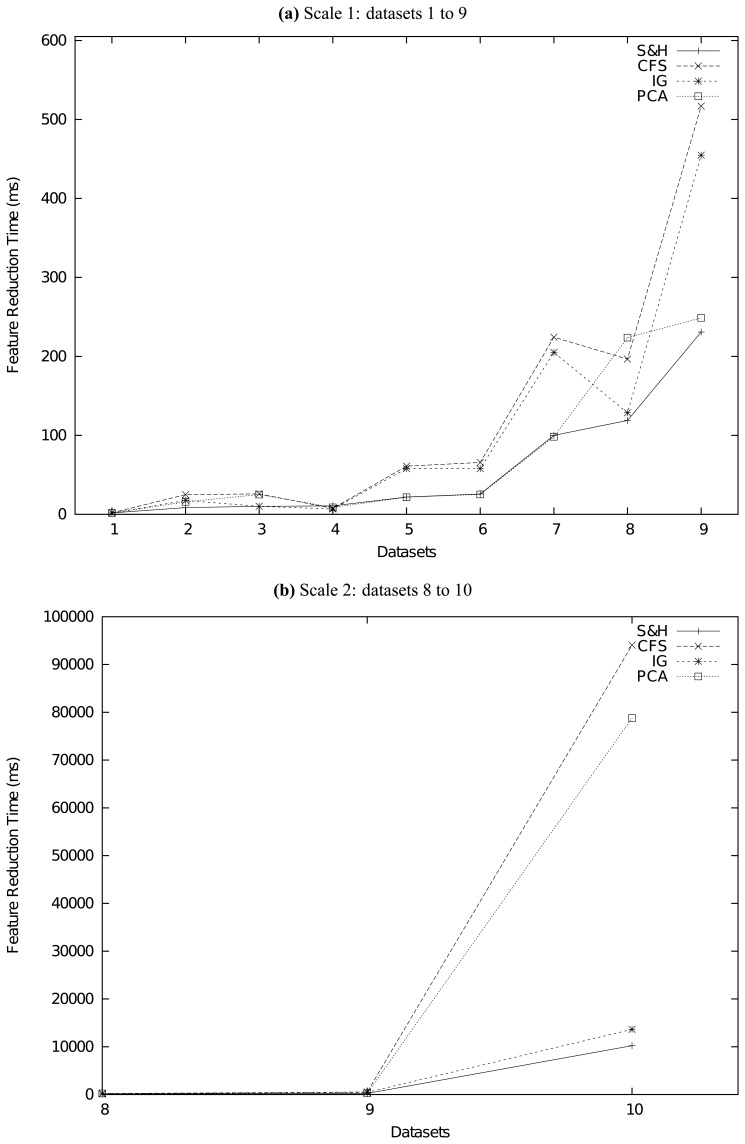
Feature reduction time comparisons.

First, let us talk about the feature reduction time. From the left part of Table 5, we see that, our method can achieve significant improvements (degradations of other methods) in most circumstances (27 times), and get just 13 draws. Furthermore, no significant improvements have been made by other methods (degradations in our method), which would be indicated by “●” in Table 5. In addition to that, it is explicit that the relief method is time-consuming. To sustain resolvability, we give a figure of feature reduction time without relief in Figure 18.

In this Figure, we illustrate feature reduction time in two scales, where the sequence numbers of datasets coincide with that listed in Table 5. Figure 17a shows the comparison plot corresponding to dataset 1–9. We see that our method is more stable than others, and its curve is almost always the lowest one. This property becomes more significant when the size and dimension of experiment dataset become larger. Figure 17b, which contains dataset 8–10, demonstrates this point clearly. From all above, we can conclude that our method is generally faster and more stable than other methods with which we make comparisons, and more suitable for high-dimensional and large scale datasets.

**Figure 19.**
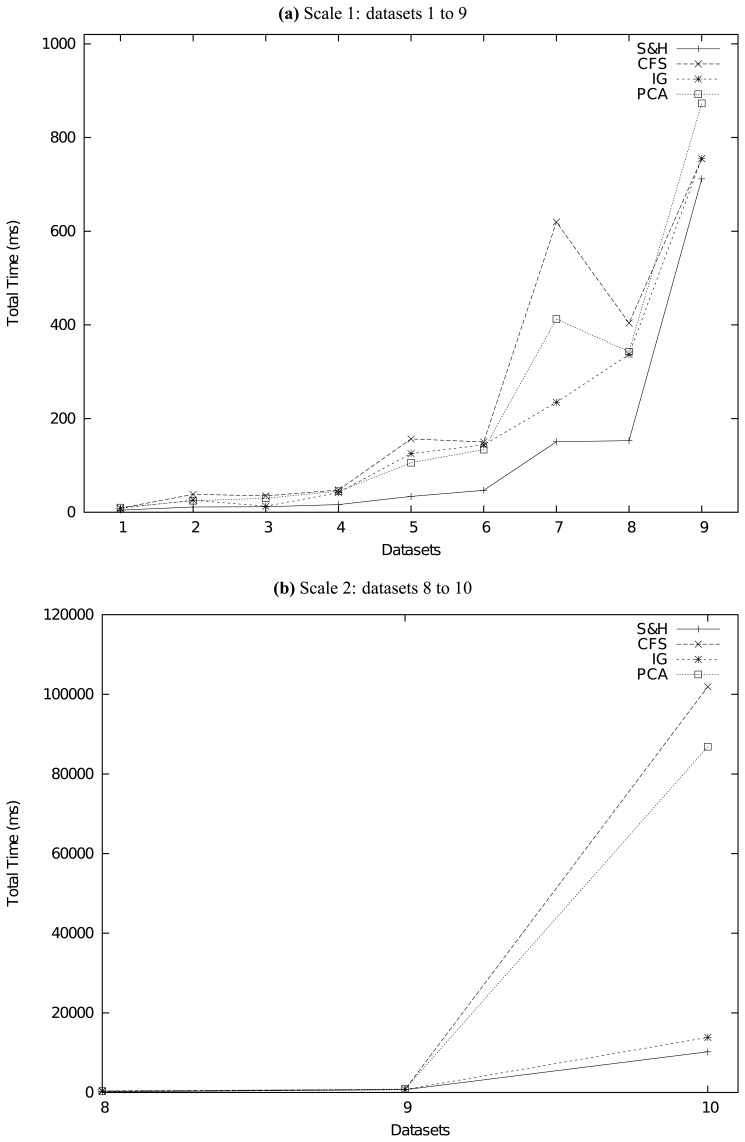
Total time comparisons. In this figure, dataset sequence numbers denote ecoli, wdbc, sonar, yeast, segment, segmentation, waveform, sensor, magic, isolet sequentially.

When we inspect the total time section of Table 5, we can confirm that our method is significantly faster than others. Except the 5 draws, our method always shows significant improvements compared with other methods. Because our method can select features not only more efficiently but also more effectively, it is prone to producing less selected features to feed the clusterers, and as a result leading to less total run time. Figure 19 illustrates this conclusion evidently. In this figure, the sequence order of datasets is different with that in Table 5, because the datasets in Table 5 are listed in the order of feature reduction time.

Next, we give the log-likelihood comparisons of feature-reduced hierarchical clusterer in Table 6. As we know, the larger the log-likelihood quantity is, the better the model fits the data [41]. Hence, we use “●” in this table to denote statistically significant degradation compared with our method. We should notice that increasing the number of clusters normally increases the likelihood, but may overfit. Therefore, to be fair, in the following experiments, we let the rankers of feature selectors retain the same number of attributes. Also note that four datasets are removed from the comparisons because their sizes or dimensions are too big to be populated into a typical computer RAM to give any experiment result. In the future, we plan to develop a distributed version of our algorithm to do more experiments on this kind of large-scale datasets. From the self-explanatory results in Table 6 we can confirm the superior performance of our method again.

Table 7 gives the similar comparisons of log-likelihood for feature reduced Simple EM clusterer. Experiment results in this table confirm the conclusions derived from Table 6 once again.

Lastly, note that although the results in Tables 6 and 7 look simple and clear, it took us really long computing time to get them, because of the inefficiency and large memory requirement of these two target back-end methods (hierarchical clusterer and simple EM clusterer), together with the huge quantities of the experiment datasets adopted in these two tables. Although we have not given the run time comparisons of experiments illustrated in these two tables because of the limitation of space, our method runs much faster than other methods. Besides, thanks to the high efficiency in design and implementation, our method can even give the experiment results when dealing with extremely large datasets, while nothing could be given by some other target methods, because of either the CPU-power or main memory limitations. Furthermore, because it is more effective and can give less selected features, the back-end methods can run much faster and have much lower limitations on main memory, thus the total speed and feasibility can be improved a lot by our method.

## 7. Conclusion

In this paper, we proposed a novel two-stage framework for feature reduction/selection. The first stage is random seeding and the second stage is uniformly partitioning-based harvest. Our new framework improved the traditional value-based evaluation and searching schema into an order-based one, which is much more effective, more efficient, and more robust. We did a series of experiments to compare our method with other state-of-the-art feature reduction methods on several real-life datasets. The experiment results confirm that our method is superior to traditional methods not only in accuracy but also in speed.

Essentially speaking, our method transforms the feature reduction problem into the outlier detection problem. Because there are a lot of state-of-the-art outlier detection methods, our framework can have plenty of variants. In this paper we only explored the uniformly partitioning-based method. This new framework is flexible for the facile integration of other outlier detection methods, which we will study in the future. Moreover, we can also adopt other seeding methodologies. In practice, because of the characteristics of outlier detection problems, our framework can achieve high tolerance of outliers in target datasets, which is an extraordinary feature of our framework.

Because of the simple and clear structure and level-based implementation of our method, it can be parallelized easily, and we will implement and study the parallel version of our S&H algorithm in the future.

## Figures and Tables

**Table 1.** The description of 10 datasets in our experiments

**No.**	**Datasets**	**Instances**	**Features**	**Classes**
1	ecoli	336	5	8
2	wdbc	569	30	2
3	segmentation	2310	15	7
4	isolet	6238	617	26
5	magic	19020	10	2
6	segment	2310	17	7
7	sensor	5456	23	4
8	sonar	208	60	2
9	waveform	5000	40	3
10	yeast	1484	8	10

**Table 2.** Squared errors for feature selected SimpleKMeans (the fewer, the better).

**Dataset**	**UnSelect (target)**	**CFS**		**IG**		**PCA**		**RLF**		**S&H**	
ecoli	142.15	142.15		142.15		139.71		142.15		124.17	●
yeast	735.58	734.83		705.61		671.66		705.61		583.64	●
sonar	476.80	116.96	●	30.09	●	26.78	●	36.00	●	20.59	●
wdbc	212.10	76.66	●	34.05	●	29.31	●	38.92	●	4.34	●
segmentation	2343.31	2111.75	●	1819.35	●	1733.75	●	1871.64	●	1577.10	●
segment	2415.10	2118.06	●	1790.06	●	1653.19	●	1800.69	●	1509.59	●
waveform	5109.59	2895.61	●	1920.29	●	1951.29	●	1986.36	●	1571.62	●
sensor	10297.99	3470.06	●	3015.37	●	1636.99	●	3634.48	●	1815.81	●
magic	5552.81	1535.06	●	1662.54	●	3014.03	●	2255.46	●	3486.68	●
isolet	144413.40	52669.72	●	6060.16	●	5654.71	●	6449.82	●	5421.02	●

● statistically significant improvement

**Table 3.** Comparisons of our method with classical feature reduction methods by squared errors of SimpleKMeans clusterer.

**Datasets**	**Target Methods**			

	**CFS**	**IG**	**PCA**	**RLF**
ecoli	●	●	●	●
sonar	●			
wdbc	●	●	●	●
yeast	●	●	●	●
segmentation	●	●	●	●
segment	●	●	●	●
sensor	●	●		●
waveform	●	●	●	●
magic	○			
isolet	●	●		●

●, ○ statistically significant improvement or degradation

**Table 4.** Comparisons of our method with state-of-the-art unsupervised feature selection methods by squared errors of SimpleKMeans clusterer.

**Datasets**	**Target Methods**				

	**FSSEM**	**CEPI**	**MCS**	**SPECTRAL**	**SIMILARITY**
ecoli	●	●	●	●	●
sonar	●				●
wdbc	●	●	●	○	●
yeast		●	●	●	
segmentation	●	○	●	●	●
segment	●	●	●	● ●	●
sensor	●				●
waveform	●	●	○	●	○
magic	○				●
isolet	●	●		●	

●, ○ statistically significant improvement or degradation

**Table 5.** Run time comparisons (the less, the better).

**Datasets**	**Feature Reduction Time (ms)**									**Total Time (ms)**								
	
	**S&H**	**CFS**		**IG**		**PCA**		**RLF**		**S&H**	**CFS**		**IG**		**PCA**		**RLF**	
ecoli	1.79	1.86		1.61		1.60		45.19	○	4.67	8.80	○	8.50	○	9.88		53.76	○
wdbc	8.45	24.95	○	17.56	○	15.66	○	507.98	○	11.23	38.30	○	25.82	○	24.76	○	516.19	○
sonar	10.13	25.88		10.12		25.04	○	145.69	○	11.67	34.98	○	12.81		29.91	○	148.23	○
yeast	10.62	7.50		6.68		8.80		928.76	○	16.28	47.58	○	41.99	○	45.05	○	962.06	○
segmentation	21.88	61.06	○	57.86	○	21.65		4219.27	○	46.72	150.10	○	143.97	○	133.80	○	4292.17	○
segment	25.68	65.66	○	57.81	○	25.22		4609.78	○	33.87	156.54	○	125.21	○	105.82	○	4678.81	○
sensor	100.02	224.30	○	205.02	○	98.17		34498.79	○	152.80	403.86	○	336.86	○	342.87	○	34624.76	○
waveform	118.89	196.46	○	128.53	○	223.67	○	49446.54	○	150.47	619.43	○	234.68	○	412.53	○	49597.85	○
magic	230.87	516.89	○	454.70	○	248.80		191700.68	○	711.69	754.85		755.00		872.79		192034.83	○
isolet	10206.08	94062.01	○	13641.42	○	78800.67	○	1126765.89	○	10237.04	101905.68	○	13830.86	○	86849.56	○	1126904.47	○

Our S&H is the comparison target; ○ means statistically significant degradation compared with S&H

**Table 6.** Log-likelihood comparisons of feature-reduced hierarchical clusterer.

**Dataset**	**S&H**	**CFS**		**IG**		**PCA**		**RLF**	
segmentation	–47.18	–59.31	●	–59.29	●	–59.27	●	–59.29	●
segment	–42.65	–55.01	●	–55.01	●	–55.01	●	–55.01	●
ecoli	2.58	0.13	●	0.15	●	0.20	●	0.15	●
wdbc	6.83	5.18	●	5.18	●	5.18	●	5.18	●
yeast	8.08	6.64	●	6.64	●	6.57	●	6.64	●
sonar	64.94	64.01	●	64.01	●	64.01	●	64.01	●

● statistically significant degradation compared with our method

**Table 7.** Log-likelihood comparisons of feature-reduced simple EM clusterer.

**Dataset**	**S&H**	**CFS**		**IG**		**PCA**		**RLF**	
segmentation	-55.99	-59.66		-59.26		-55.84		-59.29	
segment	-52.17	-55.45		-55.08		-51.43		-55.32	
ecoli	2.24	1.37	●	1.55	●	1.55	●	1.56	●
yeast	7.35	6.89		6.89		6.89		6.89	
wdbc	8.15	5.18	●	5.02	●	6.30	●	5.15	●
sonar	68.31	65.21	●	65.17	●	67.35		65.19	●

● statistically significant degradation compared with our method
